# Population Dynamics of Metastable Growth-Rate Phenotypes

**DOI:** 10.1371/journal.pone.0081671

**Published:** 2013-12-02

**Authors:** Lindsay S. Moore, Elad Stolovicki, Erez Braun

**Affiliations:** Department of Physics & Network Biology Research Laboratories, Technion, Haifa, Israel; Tel Aviv University, Israel

## Abstract

Neo-Darwinian evolution has presented a paradigm for population dynamics built on random mutations and selection with a clear separation of time-scales between single-cell mutation rates and the rate of reproduction. Laboratory experiments on evolving populations until now have concentrated on the fixation of beneficial mutations. Following the Darwinian paradigm, these experiments probed populations at low temporal resolution dictated by the rate of rare mutations, ignoring the intermediate evolving phenotypes. Selection however, works on phenotypes rather than genotypes. Research in recent years has uncovered the complexity of genotype-to-phenotype transformation and a wealth of intracellular processes including epigenetic inheritance, which operate on a wide range of time-scales. Here, by studying the adaptation dynamics of genetically rewired yeast cells, we show a novel type of population dynamics in which the intracellular processes intervene in shaping the population structure. Under constant environmental conditions, we measure a wide distribution of growth rates that coexist in the population for very long durations (>100 generations). Remarkably, the fastest growing cells do not take over the population on the time-scale dictated by the width of the growth-rate distributions and simple selection. Additionally, we measure significant fluctuations in the population distribution of various phenotypes: the fraction of exponentially-growing cells, the distributions of single-cell growth-rates and protein content. The observed fluctuations relax on time-scales of many generations and thus do not reflect noisy processes. Rather, our data show that the phenotypic state of the cells, including the growth-rate, for large populations in a constant environment is metastable and varies on time-scales that reflect the importance of long-term intracellular processes in shaping the population structure. This lack of time-scale separation between the intracellular and population processes calls for a new framework for population dynamics which is likely to be significant in a wide range of biological contexts, from evolution to cancer.

## Introduction

The Neo-Darwinian framework of evolution has made a clear separation between the sources of variation and the processes of selection in shaping the structure of populations. Within this framework, previous research on asexually reproducing populations has focused primarily on random mutations and natural selection as the dominant forces in evolution [1]–[9]. In large populations, changes in viability and growth-rate are assumed to be driven solely by random changes in genotype, occurring on long time-scales separated from the physiological time-scale of reproduction [1], [10]. Thus, under constant environmental conditions, the individual division rate is assumed to be stably inherited along lineages and changes only due to random mutations [7]. The fitness of an asexual population is then simply the population-average growth rate [3], [7], [8], [11], [12] and selection results in a steady increase in population fitness with time. Following the Neo-Darwinian paradigm of mutation-selection, experiments on evolving populations of microorganisms have focused on the long-term fixation of mutations [1], [4], [6], [10], [13], [14], successfully describing the long-term dynamics seen in these populations. In the limit of large populations, effects such as clonal interference and multiple mutations can enrich the dynamics of fixation. Measurements of clonal interference in large populations of yeast have shown fitness effects that occur on ∼100 generation timescales [4], [6]. The dynamics of fixation of genetic mutations are easy to recognize: because only beneficial mutations are selected for, the average fitness of the population increases monotonically. Additionally, the timescales of fixation are determined by many factors such as the population size, the rate of beneficial mutations and the fitness advantage they confer. In the yeast *S. cerevisiae* the time-scales of fixation in large populations have been documented to be longer than 100 generations [4], [15].

Notwithstanding the success of the Neo-Darwinian framework, it is important that the intermediate time-scales in which the processes of selection and variation coexist do not go unstudied. Recent research has uncovered a rich spectrum of processes that lead to phenotypic variability over a wider range of time-scales than those caused by genetic fixation. These include heritable epigenetic phenotypes that do not show underlying genotypic changes [16], [17]. Thus, along the path to fixation, population dynamics involve a wealth of processes beyond genetics that lead to inherited phenotypic variability, covering the range of time-scales that span from fast physiological processes that occur within one generation, to extremely slow mutations that can take hundreds of generations to become fixed [18]. Little information exists on the dynamics of processes that lead to adaptation and the intermediate states of evolving phenotypes enabled by cellular plasticity, some of which are known to be stably inherited over many generations [19], [20]. A thorough understanding of these dynamics is required to predict evolution outcomes and understand heterogeneous populations. Recent studies in eukaryotes have emphasized the importance of inherited epigenetic processes in shaping phenotypes, including growth rate, that occur on time-scales similar to reproduction, and can be less stable than genetic mutations [16], [21]–[23]. However, experiments studying the effects of epigenetic phenotype changes and inheritance on population dynamics are still lacking.

In this paper, we present measurements of population dynamics in genetically rewired yeast faced with a severe, unforeseen, regulatory challenge [24]. We have shown before that cells adapting to overcome this challenge exhibit a spectrum of intracellular processes on a wide range of time-scales, thus giving us an opportunity to study novel aspects of population dynamics [24]–[29]. The essential gene *HIS3* — required for histidine synthesis — was deleted from its native position in the genome and replaced under the exclusive regulation of the GAL system, responsible for galactose utilization. In medium lacking histidine, upon a switch from galactose to glucose, the GAL system is highly repressed and the rewired cells encounter a severe challenge. We have shown before that such populations adapt to grow exponentially within fast time-scales of ∼10 generations and this adaptation is inherited at the population level [24], [25]. Moreover, detailed experiments have shown that the recovery of the population is not due to selection of a rare subpopulation; more than 50% of naïve cells spread on agar plates made with glucose medium and lacking histidine will develop into a colony of cells able to grow exponentially, similar to wild-type cells, within two weeks of plating [24]. Moreover, the fraction of cells that can grow into adapted colonies on agar plates was shown to actually initially decrease with time from their first encounter with the glucose medium [25]. Gene expression measurements have shown that adaptation was accompanied by a large-scale genomic response [28], forming temporal patterns involving hundreds of genes and reflecting dynamics of highly-correlated cells within the population [29]. Our previous work identified multiple trajectories leading to adaptation. While in some of the cases mutations can be identified in the adapted populations, we have shown that adaptation requires additional processes beyond a mere change in DNA sequence [26]. Moreover, based on detailed analysis we concluded that genetic changes could not account for the entire spectrum of adaptation solutions [25], [26].

In the experiments discussed here, a large population of adapting cells is grown in constant environmental conditions for hundreds of hours and yet, as shown below, even after exponential growth resumes we measure a very broad distribution of single-cell growth rates (σ/μ is about three times that measured for wild-type cells). These broad distributions persist for very long durations of greater than 100 generations, indicating that subpopulations with a wide range of growth rates coexist for long periods. The mean growth rate also does not increase monotonically, as is expected with simple selection, but instead continues to fluctuate throughout the experiment. Of note is that the fluctuations include plateaus of long duration and decreases in the average growth rate, indicating an overall decrease in the population fitness. Additionally, we measure other phenotypes in the population, including the HIS3p content, and the ability of cells to grow exponentially. These phenotypes also fluctuate significantly, throughout the duration of our experiment, on time-scales of several generations that cannot be explained by the fixation of a new genotype. These experimental results show that the phenotypic state of cells in a constant environment can be metastable, exhibiting variability on time-scales that broaden our understanding of population dynamics during regulatory evolution.

## Results

**Figure 1:**
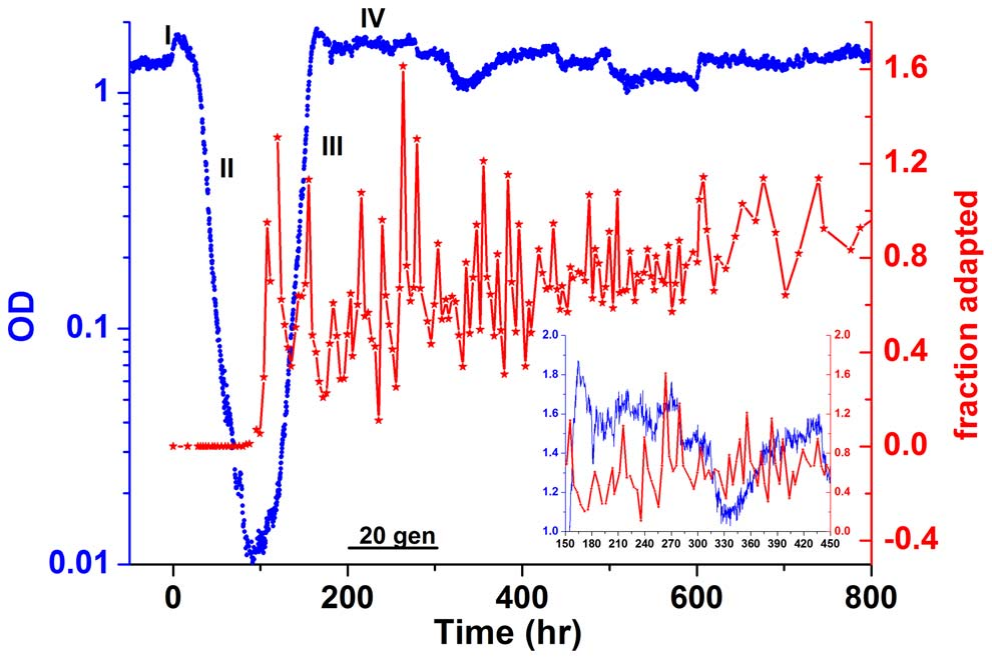
Chemostat population dynamics. The blue trace shows the typical population density (measured by the OD at 600 nm) as a function of time for our rewired cells grown in a chemostat, upon switch from galactose medium to glucose medium lacking histidine (glu-his) at t = 0. Note the logarithmic scale. The four phases of the dynamics are marked I-IV. The red trace shows the number of cells that are able to grow a visible colony within 3 days after plating on glu-his agar plates (“fraction adapted”) relative to the number of colonies grown on rich medium plates (and thus can be larger than 1). Inset: a subset of the blue and red curves, focusing on the time between 150–450 hrs after switch to glucose. Bar: 20 chemostat generations.

Measurements of the cell density of a chemostat culture of rewired cells show the global dynamics of adaptation and the population-level response to the genome rewiring challenge (Fig. 1, blue line). The dynamics following a switch from galactose to glucose are characterized by four phases: I) an exponential increase in cell density; II) an exponential drop in density due to a reduction in the average growth-rate; III) an exponential increase in density reflecting a growing fraction of the population resuming high growth rates; and IV) a more stable, high-density state indicative of an adapted population [24]. These phases have the hallmarks of an evolutionary process. Nevertheless, adaptation takes place without selection and on time-scales (∼10 generations) that are much faster than those observed in other known laboratory evolution experiments [1], [6], [10], [25], [30]. At first glance, the population seems fully adapted to the glucose medium in phase IV. Surprisingly, a closer look reveals that although the population as a whole is able to sustain a qualitatively high cell density, it exhibits significant fluctuations both in its average behavior as well as in single-cell phenotype distributions. During phase IV, the cell density fluctuates, indicating that the average yield, and thus the average growth-rate and cellular metabolism, fluctuated on long time-scales of many generations (see Fig. S1 for analysis of these fluctuations and their significance in comparison with the behavior of a wild-type strain). In that same time-period, samples of cells were taken from the chemostat at 3-hour intervals and spread on agar plates made with a glucose medium similar to that feeding the chemostat. The number of cells that were able to grow visible colonies on the agar plate represents the fraction of cells exhibiting a stable, adapted phenotype (see Methods) and reaches 100% at the beginning of phase IV (Fig. 1, red line). Surprisingly, this seemingly fully-adapted population then exhibits fluctuations in the fraction of adapted cells, indicating that the adapted phenotype is not stably inherited [25]. Note that the fluctuations in the adapted phenotype are significant; there are instances in which the fraction of adapted cells in the population drops to low values of ∼10%. These measurements show that while the average population growth is exponential, new cells are continuously being born in the chemostat that are not able to stably propagate the adapted phenotype in glucose and thus to grow a colony on an agar plate. The fluctuations in the fraction of adapted cells seem to decay on a time scale of ∼100 generations, converging to values close to 100%.

**Figure 2:**
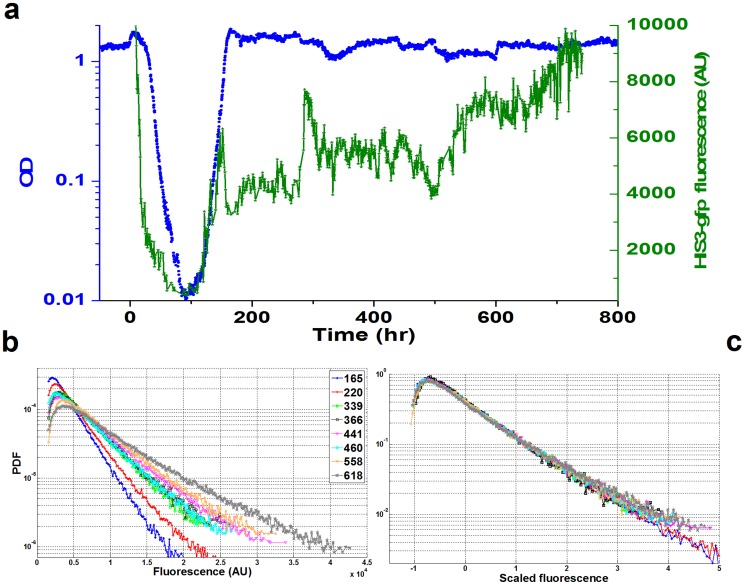
HIS3-GFP dynamics in a chemostat population. (a) The average *HIS3*-GFP fluorescence of the chemostat population (green) as a function of time for the same experiment as in Fig. 1. The population-average fluorescence was extracted from statistics over single-cell measurements utilizing our home-made cytometer online with the chemostat. The protein content fluctuates by up to a factor of two, hundreds of hours into phase IV. The optical density is shown in blue for reference. (b) Distributions of the single-cell *HIS3-GFP* for several time-points during phase-IV. (c) All the distributions from (b) collapse to a single curve when subtracting the mean and dividing by standard deviation of each distribution.

Throughout phase IV there are also striking dynamics in the HIS3p distribution in the population. Using a strain of rewired yeast with HIS3p tagged with GFP, we measured the dynamics of *HIS3* protein content of individual cells utilizing a home-made flow cytometer on-line with the chemostat. Figure 2a shows that the average HIS3-GFP content of the population fluctuates with time, by up to a factor of two, hundreds of hours into phase IV (Fig. S2 shows the stability of the home-made cytometer). Figure S3 shows similar fluctuations in a repeated experiment. These dynamics are consistent with our previous measurements showing slowly-varying fluctuations in the mRNA levels in similar population experiments [29]. As shown before, the precise pattern of expression was irreproducible between repeated experiments.

Single-cell measurements show that HIS3p is broadly distributed within the population (Fig. 2b). Previously, variations in phenotype in clonal populations have broadly been categorized as “noise”; resulting from molecular and cellular stochastic processes [31], [32]. Although epigenetic processes could in principle lead to traces of trans-generation correlations in the fluctuations, implicit in that categorization is a time-scale reflecting the molecular gene expression processes on the order of, or less than the cell division time. The mean expression of HIS3p shown in Figs. 2a and in the distributions of Fig. 2b, exhibit relaxation dynamics on time-scales on the order of several generations, much beyond previous measurements of correlated fluctuations in microorganisms [33]. Furthermore, although the mean expression varies as a function of time, the shape of the single-cell protein distributions was preserved throughout phase-IV. Remarkably, Fig. 2c shows that all the distributions of Fig. 2b collapse to a single curve by subtracting the mean and dividing by standard deviation. Figure S4 shows that this scaling preserves the shape of the distributions for the entire range of phase IV. This type of universality was discussed in our previous publication [34] which concluded that the shape of the distributions cannot be determined by a specific intracellular process. Moreover, consistent with the measurements in ref. [34], the variance throughout phase-IV is a quadratic function of the mean (Fig. S5), showing that throughout the adaptation process a single population-average variable determines the gene expression distribution. Thus, a universal, single-parameter scaling of the essential HIS3p distribution indicates that the population as an entity plays a critical role in the dynamics; the wide protein-content distribution among cells does not result from intracellular noise fluctuations in gene expression but rather from collective population processes [29]. Note that the slowly-varying population-average fluctuations, lasting many generations as observed here, reflect a cooperative process between cells. Thus, if the expression level would fluctuate independently in each cell, these fluctuations would be averaged out in our large populations of 10^10^ cells. Thus, comparison with previously published results shows that the gene expression in the case of an absolutely essential gene in an adapting population is not fundamentally different from other (essential or non-essential, highly regulated or constitutive) genes in cell populations grown under a wide range of conditions [34].

So far we have shown measurements of the population-average density, the fraction of adapted cells in the population, and the single-cell protein distributions, which all indicate that the population exhibits a highly-dynamic structure during phase-IV. Next, we present single-cell measurements that show that the ability to grow and growth-rates themselves are not stably inherited. Single-cell growth rate is an important parameter in evolutionary and population biology as an indicator of cellular fitness relying on the functionality and coordination of cellular subsystems. We used a microscopy assay to measure the instantaneous growth rate of single cells throughout phase-IV. Cells from batch culture were switched from galactose to glucose and propagated throughout the adaptation phases by serial dilutions. Large samples of these batch cultures were then measured to gain a broad statistical view of the single-cell growth-rate distribution. We have shown before that the adaptation dynamics including the four phases observed in chemostat experiments, are reproduced in batch cultures [25]. Figure S6 also shows that, similar to the chemostat experiment of Fig. 1, the fraction of adapted cells in the batch cultures fluctuates over long durations during phase IV of the dynamics. Importantly, by growing the populations in dilute batch cultures with excess nutrients for the entire growth period, we can ensure that there are no stressed cells in the population due to nutrient limitation. Note that the propagating populations remained large throughout the experiments and never went through bottlenecks [35] (see Methods). For each experiment, at different time points throughout the propagation, the instantaneous growth rate of hundreds of cells were measured using the method described in reference [36] (Methods). Each measurement was limited to a short duration of ∼3 generations, allowing us to capture the phenotypic variability in the population while ensuring a single-exponent growth curve (Rsq>0.95; Fig. S7). Thus, this instantaneous growth-rate determines a “local” variable, which in the population context varies on longer time-scales. Colonies that were measured not to be growing exponentially were analyzed separately, and are discussed below.

**Figure 3:**
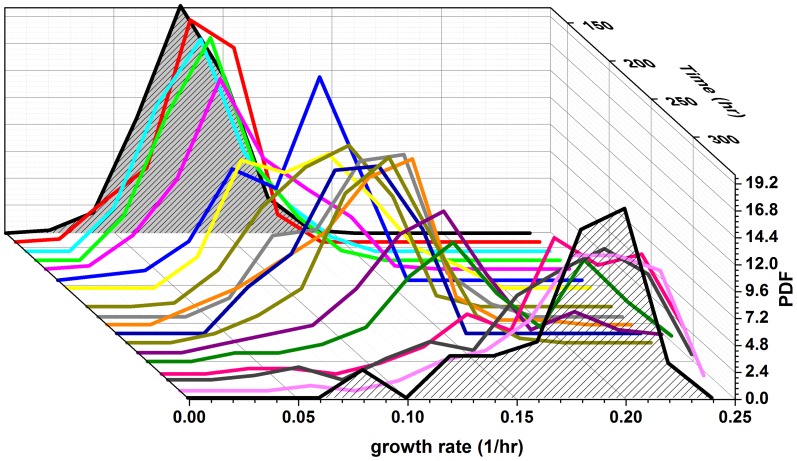
Single-cell, instantaneous growth-rate distributions. Histograms of the growth-rates plotted as a function of time. The instantaneous growth-rates were estimated from time-lapse microscopy measurements (Methods). The mean division time of the first distribution is 8.4 hours.

**Figure 4:**
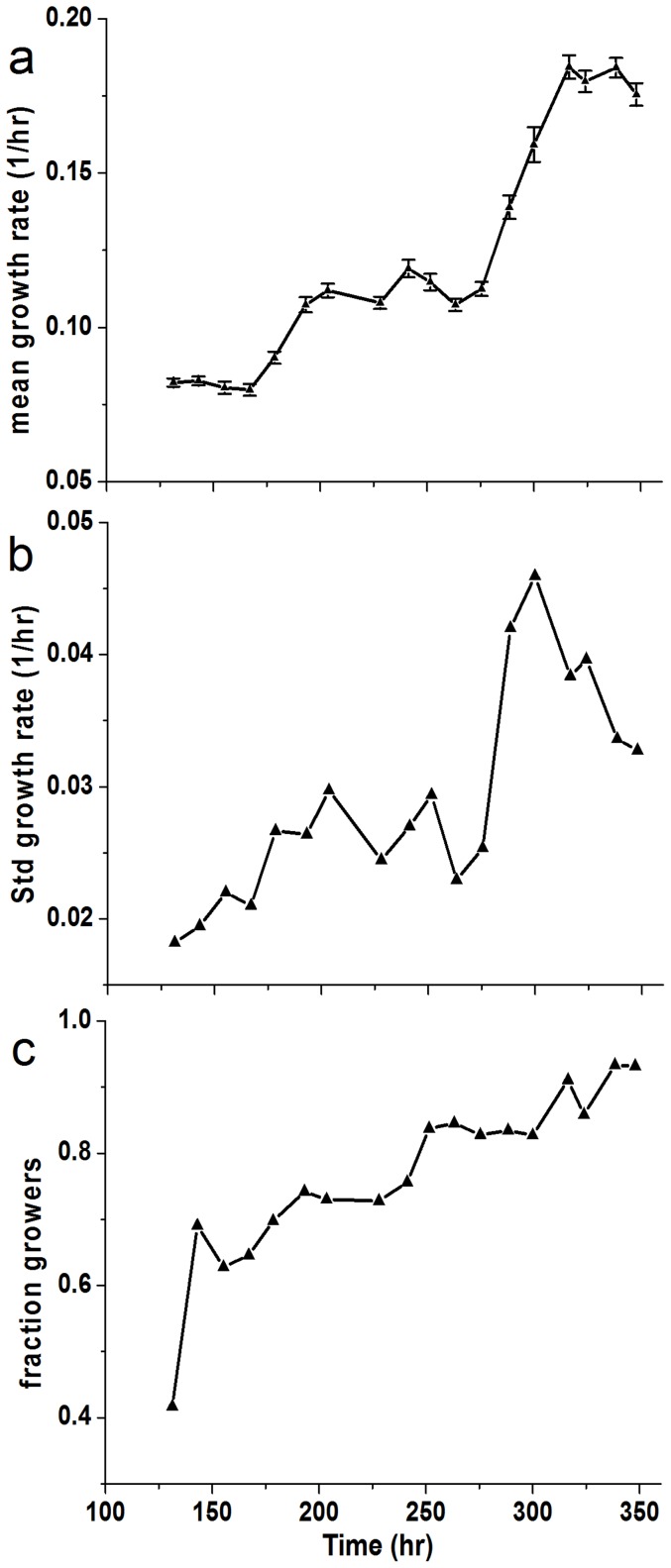
Mean and std of growth-rate and viability. (a) Mean growth-rate extracted from the histograms in Fig. 3. Note the plateaus in the mean growth rate that persist for many generations, followed by decreases and sharp increases. Error bars are the standard error of the mean. (b) The standard deviation of growth-rates computed from the same distributions leading to the mean values presented in (a). (c) Fraction of the population that was measured to be growing exponentially in the same time points as (a). Although the population culture was diluted every 12 hours, the fraction of cells that grew exponentially did not approach 1 for hundreds of hours.

**Figure 5:**
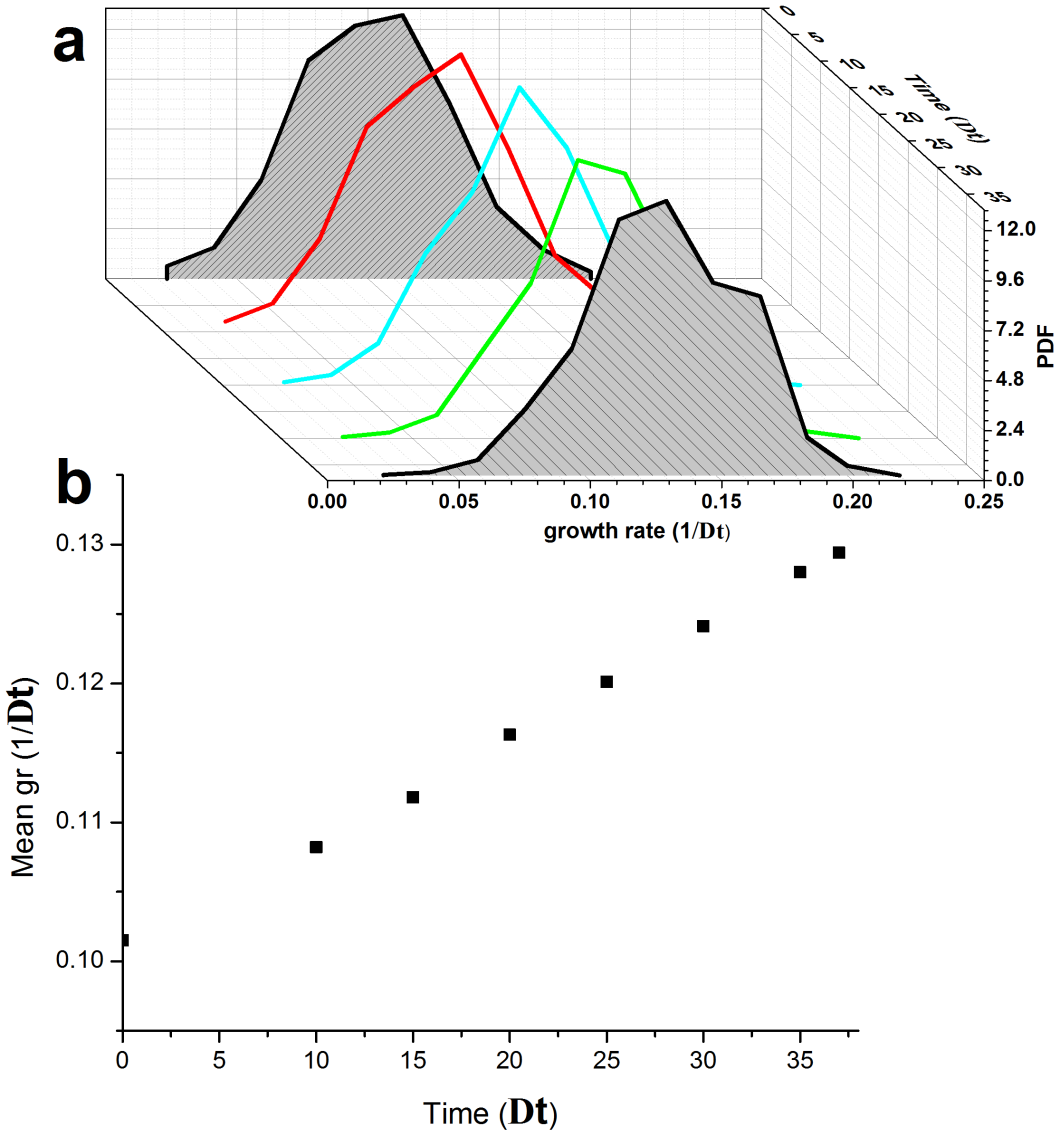
Monte-Carlo simulations of an evolving population. (a) Growth-rate distributions as a function of propagation time, computed from a Monte Carlo simulation of the evolution of a population with a stably inherited growth-rate in a lineage across generations. There are 1000 cells at the initial time point serving as the seeds for the evolving lineages. In each lineage the growth-rate is extracted from a Gaussian distribution. (b) The mean growth rate in the simulation increases lineally as a function of time. The mean growth rate changes by ∼30% within ∼5 generations. The growth-rate is measured in units of the inverse simulation time.

Figure 3 shows the histograms of the instantaneous growth-rates from one such experiment as a function of time in glucose (repeated experiments are shown in Fig. S8). The growth-rate distributions are quite broad (σ/μ∼0.3) throughout the entire measurement period, which lasts >60 generations, with significant dynamics also manifested in the standard deviation (Fig. 4b). Remarkably, the population experiences long periods with almost static growth-rate distributions, followed by periods of decreasing and increasing growth-rate without exponential takeover of the fastest growing cells. Thus, there is no indication of a sub-population of fast growers dominating the population dynamics. This unusual dynamics is also manifested in the mean growth-rate extracted from these distributions (Fig. 4a). Note the non-monotonic dependence of the growth-rate and the emerging plateaus lasting for up to 25 generations. Repeated experiments show similar dynamic fluctuations (Fig. S9). Monte Carlo simulations of the expected evolution of a population exhibiting a distribution of growth-rates, assuming only simple selection and inheritance of growth-rate in lineages, verify our assertion that the fastest growers should be expected to take over monotonically within several generations (Fig. 5). Simulations that include inheritance of growth-rates cannot reproduce the non-monotonic changes in the population-average growth-rate for large populations, in particular plateaus on time scales of ∼25 generations and periods of decrease as observed in our experiments (Fig. 4a and Fig. S9a). In contrast, our measurements reveal the co-existence of fast and slow growers over extended durations of almost 100 generations.

More dramatically even is the coexistence of non-growing cells during this period. The same microscopy samples were used to measure the fraction of exponentially growing cells in the population as a function of propagation time (Fig. 4c; repeated experiments, Fig. S10). Although the fraction of cells able to grow exponentially is generally increasing and eventually converges to 100% after extended durations, since the populations were serially diluted, the persistence of non-growing cells for such long durations is remarkable. It shows that although the population as a dynamic entity had adapted, the ability of cells to grow exponentially is not stably inherited. This is in contrast to wild-type cells, which have a constant 99% fraction of exponentially growing cells, showing a narrow growth-rate distribution (σ/μ∼0.1 compared with σ/μ∼0.3 for rewired cells in phase IV-Fig. 3) with an average that barely changes with time (Fig. S11). These results prove again that the fastest growing cells do not take over our adapting population; in fact, subpopulations of non-growers, slow growers and fast growers coexist, forming a dynamic, continuous spectrum of growth phenotypes within a proliferating population.

## Discussion

The genetically rewired cells in our experiments are faced with a severe, unforeseen challenge upon medium switch from galactose to glucose. Although after an adaptation period, the population as a whole can sustain a high cell-density with exponential growth at rates similar to wild-type cells, the individuals composing the population are actually in metastable phenotypic states. Such meta-stability is the hallmark of epigenetic processes [16], [37]. It shows that from the single-cell perspective, the adaptation process is not completed even when the population-average dynamics seem relatively stable. An increasing body of research indicates that the phenotypic state of a cell, its metabolism, morphology and growth-rate, are determined by dynamical processes resulting from the complex interplay of numerous genes through protein-DNA and protein-protein interactions. Growth rate is an integrated outcome of metabolism and gene expression that is particularly significant in determining the fitness of the cell to the environmental conditions and its competition with other individuals. It is the intracellular processes of gene expression and metabolism that transform genotypes into phenotypes. This transformation is a dynamical process sensitive to the interaction of the cell with its environment and involving a wide range of time-scales. The complexity of intracellular processes ensures that the connection between genotypes and phenotypes is far from being a simple one-to-one mapping. The results presented in this paper show that, at least under certain conditions, the direct mapping of a mutation to fitness is not always an adequate basis for modeling population dynamics. From the experimental view point, while the genetics approach to population dynamics has been successful at describing evolution on long time-scales, population dynamics involve a wealth of processes beyond the fixation of beneficial mutations and thus require measurement of the intermediate phenotypes throughout the evolutionary process. Thus, the high temporal resolution measurements of the population structure presented above open a vista on evolutionary processes beyond the fixation of mutations.

From a broader perspective, our results paint a picture of an intracellular exploration process [28], [29] rather than convergence to a rare, fit variant. In other words, the physical process of gene interactions lead to dynamics in a high-dimensional phase-space spanned by the concentration of expressed proteins [38]. The phenotypic state of a cell reflects the stabilization of these dynamics. Outside of cases in which the phenotypic state is determined by rigid “hard-wired” interactions (e.g. when cells encounter a familiar stress or a change in environment), the stabilization of a state in this complex phase-space is determined by an exploratory process involving numerous metastable phenotypic states and very long convergence time-scales [29]. The significance of the effect of such complex processes on the population dynamics should not be underestimated. Indeed, our results presented above show the coexistence of a wide spectrum of growth-rate phenotypes, lasting very long durations for an exponentially proliferating population in a constant environment.

Phenotypic variability, including epigenetic inheritance, also plays an important role in the framework of bet-hedging in which variable populations show increased fitness compared with homogeneous populations in fluctuating environments [39], [40]. However, unlike the concept of bet-hedging, in which population variability maintained in a benign environment serves to rescue the population upon a switch to harsh conditions [36], [41], [42], our phenotypic variations represent exploration toward stabilizing a relaxed, adapted state all within a constant environment. Note that in the framework of bet-hedging, the emerging spectrum of phenotypes remains neutral as long as the environment is stable. In particular, large variability in growth-rates cannot be maintained in a constant environment due to selection. By contrast, exploratory dynamics as observed in our experiments support a wide variability in growth rates over extended durations. The dynamics determining this spectrum of observed phenotypes are the result of overlapping time-scales between the intracellular processes determining the phenotypes of individuals and the frequency distribution of different states determined by population selection forces. This mixing of processes between two levels of organization—individuals and the population—calls for significant extensions of existing frameworks of population dynamics in evolution and other biological contexts such as cancer [43], [44].

## Materials and Methods

### Strains

Experiments were carried out using three different strains of S. cerevisiae, all with *HIS3* under exclusive regulation of pGAL1. Cloning was done by standard methods and was confirmed by fragments analysis and/or by direct sequencing. Transformation was done with the lithium acetate method:

- haploid yeast strain YPH499 (Mata, ura3–52, lys2–801, ade2–101,trp1-Δ63, his3Δ200, leu2Δ1) carrying the plasmid pESC-LEU (Stratagene) containing the pGAL1-pGAL10 divergent promoter with HIS3 under pGAL1 as described in [24]. (Figs. 3,4, S9, S10 black and red)
- YPH499 with the pGAL1- GFP-HIS3 cassette (N terminal) integrated into the Leu2 locus. (Figs. 1, 2, S9, S10, blue and green)
- YPH499 with GFP/RFP integrated under ADH1 promoter at the Ura3 locus [36] carrying the plasmid pESC-LEU. (Figs. S9, S10, orange)

### Chemostat

Cells were grown in a home-made chemostat with a 135-ml working volume and temperature controlled at 30°C, as described in [24] with a dilution rate of 0.18-hr^−1^ (controlled by a digital peristaltic pump; Ismatec). To verify that the dilution rate was constant throughout the experiment, the pump rate was measured offline using the same apparatus at the beginning and end of the experiment to within 1-mL/hr. Additionally, the media consumption was constantly monitored. The chemostat was inoculated with cells from a single colony from an agar plate, grown to exponential phase in synthetic dropout medium lacking histidine and leucine with standard amino acid supplement and 2% of either high grade pure galactose or pure glucose as a sole carbon source. Growth in the chemostat was limited by the concentration of the amino acid supplement (verified by increase in steady-state OD of the chemostat culture with increase in amino acid concentration). Throughout the experiments, the sugar (either galactose or glucose) is always in excess (maximal consumption of the cells is 25% of the sugar supplied). Medium was: 1.7-g/liter yeast nitrogen base without amino acids and ammonium sulfate, 5-g/liter ammonium sulfate, 1.4-g/liter amino acid dropout powder (without tryptophan, histidine, leucine, and uracil; Sigma, St. Louis), 0.006-g/liter L-tryptophan, and 0.003-g/liter uracil. Temperature was maintained at 30°C and the culture was continuously mixed while air was pumped into the growth chamber. The OD at 600 nm was measured by a dedicated spectrophotometer (Ocean Optics, Dunedin, FL) coupled optically to a flow channel at the chemostat outlet (light source: tungsten lamp).The chemostat population at OD = 1 contains about 10^9^–10^10^ cells and the generation time is the chemostat dilution time times ln(2), which was about 5 h.

### Flow cytometer online with the chemostat

A home-made flow cytometer allowing real-time single-cell multi-color fluorescent measurements, online with the chemostat was constructed. The device injects cells from the running chemostat directly into a flow chamber which in turn hydrodynamically focuses the injected cells into a single-file toward the detection zone. An optical setup collects the laser (Sapphire 488-30, Coherent) light scattered from single cells and the measured signals are acquired by a data acquisition board connected to a computer (PD2-MF-16-3M/12H, United Electronic Industries). Home-made software controls sample injection into the flow chambers, washing and extracting pulse information (height, area and width) for fully automated measurements over extended periods of the experiment. We measure simultaneously forward scattering (FL488-10, Thorlabs), side scattering (FL488-10, Thorlabs. H9656 Hamamatsu photonics) and fluorescent signals (ET520/40, Chroma. H9656-01, Hamamatsu). As part of the quality control of the home-made setup, aliquots from the chemostat were measured in parallel using a commercial flow cytometer and compared to the measurements from the home-made flow cytometer. Figure S2 shows the accuracy and stability of the cytometer. Figures S2 a,b show the comparison of our cytometer data with a commercial flow cytometer (BD LSR-II Analyzer), with agreement between the data sets of >95%. Deviations at low fluorescence are due to differences in the sensitivity and dynamical range between the machines. Figure S2c shows the stability of the measurements by comparing the HIS3p-GFP distributions of a chemostat population in galactose medium over a period of 100 hours (∼20 generations). The distributions shown in Fig. 2 and Fig. S4 were measured on large samples, containing 40,000–250,000 cells depending on the chemostat population density.

### Batch culture

Batch cultures were grown in medium similar to the chemostat medium, comprised of 1.7-g/liter yeast nitrogen base, 5-g/liter ammonium sulfate, 1.4-g/liter amino acid dropout powder. 1x amino acid concentration medium contains 0.4-g/liter L-tryptophan, and 0.2-g/liter uracil. In our measurements, strain 1 used 0.25X L-tryptophan and uracil, strain 2 used 0.15X L-tryptophan and uracil, and strain 3 used 0.15X L-tryptophan. These amino acid concentrations were chosen to allow more direct comparison between chemostat and batch measurements. Strain 2 was the same yeast strain that was used in the chemostat.

The batch culture was diluted every 12 hours to maintain OD<1.0 and ensure that the cells always experienced an excess of nutrients. Previous measurements have shown that exponential growth persists in this medium until OD>3.0. During all dilutions, care was taken to transfer a large fraction of the population to avoid population bottlenecks that would skew our results. Specifically, depending on growth rate, batches were diluted by a factor of between 1∶10 and 1∶50 every 12 hours, never transferring fewer than 10^5^ cells.

Populations adapted in serially-diluted batch cultures exhibit variability in their adaptation times to glucose, similar to chemostat populations as shown in the experiments in Fig. 2 of ref [25]. Measurements plotted in Figs. S8, S9, S10 show a time axis after transfer to glucose. The time of the first reported measurement point is based on when the first cells started to grow exponentially, hence the variability in the time-axes between the different experiments.

### Microscopy assays for growth-rate measurements

The microscopy assay was similar, including all controls, to that described in [36]. Flat-bottomed 96-well plates (NUNC 167008, well area 30 mm^2^) were filled with 200-µL of 200-mg/ml Concanavalin A (Type IV, Sigma) for 2–6 h. Wells were washed once with 400-µl of distilled water immediately flung out. Coated 96-well plates were used immediately, or wrapped and stored at 4°C for use within 24 hrs. Plates were warmed to 30°C before use.

Cell batch cultures were grown to OD<1.0, then diluted 1∶10 into fresh medium. 100-µL of the diluted cell culture was pipetted into 20-mL of filtered medium, and agitated with the vortex for 30 seconds to mix and break up groups of cells. 200-µL of that batch was then pipetted into each of the prepared wells. An adhesive plate sealer (Edge Bio Systems #48461) was used to seal the plate, and the plate is spun at 360-g for 2-minutes at 30°C. Bright-field images were captured on a Zeiss Axio-Observer inverted microscope fitted with an incubator maintaining stage temperature at 30°C, and a fully automated ASI stage, and imaged using a 10× air objective with a 9 mm working distance. An image was captured per well (near the center, but optimized for cell spacing and lack of debris) every one hour for 12–24 hours, depending on the measured growth rate in the last plate (very slow growth required longer imaging sessions to estimate the growth rate).

### Image analysis

Bright-field images were analyzed using Matlab image processing tools and specially written colony-tracking software. The area of each colony in each frame of the movie was measured using edge-finding algorithms performed on filtered raw images. Figure S12a shows a typical microscope image with the black line surrounding each colony marking the edge used to determine the colony area. The colonies were tracked from one frame to the next using centroid correlation. The area vector for each colony was fitted to an exponential curve using an automated curve-fitting routine. Fits with an Rsq>0.95 were automatically accepted to the histogram. All others were assessed by hand to determine if there are outliers that prevent a good fit despite exponential growth. Microscopy images were all reviewed manually to verify image quality and good fits to colony perimeters. Manual counts of cell number were compared with colony areas to verify reasonable growth rate estimates (Fig. S12b). The fits to a manual cell count and the colony area from the image analysis gave the same growth rate within the fitting error. Any colonies that were not growing exponentially, were too close to the edge of the field of view, or were growing too close to another colony were excluded from the histograms in Fig. 3 and Fig. S8. All measurements containing more than 100 colonies were included in the final histograms. To verify the statistical significance of each data set, the mean was calculated from a random set of chosen points from the distribution. Increase in the number of random points led to convergence of the mean to within the standard error, indicating that our data contained sufficient points to represent the distribution. Moreover, Fig. S13 shows representative histograms from the same experiment as in Fig. 3, with error-bars confirming the significance of the distributions. This was the smallest data set, and therefore has the largest error of all presented data.

### Plates Assay

Glucose agar plates were made with the same nominal medium as the chemostat and batch media, plus 2% agar. Rich medium agar plates were made with YPD medium plus 2% agar. For each time point samples were taken from the chemostat or batch culture and diluted to ∼2000-cells/mL and 100 µL was dispersed onto each of two YPD agar plates and three glucose agar plates. The cells were distributed uniformly on the plates by shaking with pretreated sterile glass beads. Plates were incubated at 30°C for three days to allow colonies to form. Colonies were then counted on all five plates and tabulated by plate-type. The “fraction of adapted” cells (Fig. 1) is taken to be the average number of colonies that grow per glucose plate divided by the total number of colony-forming units, as measured by the average plate count from the YPD plates plated at the same time.

## Supporting Information

Figure S1 **Chemostat population density fluctuations.** (a) The OD of the same chemostat population as in Fig. 1 of the main text, in galactose before switching to glucose at t = 0. (b) Part of phase IV from the same experiment for comparison, showing the significance of the density fluctuations. (c) A population of wild type cells switched from galactose to glucose in the same chemostat apparatus. The σ/μ marked for each region separately, are of the same order as in (a). It shows that the background fluctuations in the chemostat are significantly smaller than the ones observed in phase IV in (b).
(PDF)

Figure S2 **Stability and accuracy of homemade cell cytometer.** (a) and (b) show the comparison of our home-made cytometer data with a commercial flow cytometer (BD LSR-II Analyzer), with agreement between the data sets of >95%. (c) The stability of the home-made cytometer measurements is shown by comparing the HIS3p-GFP distributions of a chemostat population in galactose medium over a period of 100 hours (∼20 generations).
(PDF)

Figure S3 **Mean HIS3-GFP dynamics in a chemostat population.** (a) A repeated experiment to the one shown in Fig. 2a. The blue trace is the chemostat optical density as a function of time after switch from galactose medium to glucose medium lacking histidine at t = 0. Note the logarithmic scale. The green trace is the mean fluorescence measurement of HIS3-GFP. The population-average fluorescence was extracted from statistics over single-cell measurements utilizing our home-made cytometer online with the chemostat. (b) The OD in phase IV of the chemostat in (a) on a linear scale between 200 and 600 hours showing significant fluctuations similar to the ones observed in Fig. 1 of the main text.
(PDF)

Figure S4 **Scaled HIS3-GFP distributions.** Single-cell fluorescence distributions measured from the same chemostat population as in Fig. 1 during phase-IV. All distributions have been scaled by subtracting the mean and dividing by the standard deviation, causing them to collapse onto a similar shape. Note that the mean fluorescence value from this same time period (Fig. 2a) has dynamic fluctuations by more than a factor of three.
(PDF)

Figure S5 **Variance vs. Mean from HIS3-GFP distributions.** Scatter plot of the variance fluorescence versus the mean extracted from single-cell fluorescence distributions for the same populations of cells as in Fig. 2, for the entire range in phase IV as in Fig. S4. The variance is a quadratic function of the mean: red curve, best fit y = −5.39+1478 x+0.48 x^2^.
(PDF)

Figure S6 **Fraction of adapted cells in a batch culture.** The number of cells that are able to grow a visible colony within 3 days after plating on glu-his agar plates (“fraction adapted”) relative to the number of colonies grown on rich medium plates from serially-diluted batch culture in phase IV, after the switch from galactose to glucose. Comparison with the red curve in Fig. 1 shows qualitatively similar fluctuations in the fraction of adapted cells in a chemostat culture and in batch cultures throughout phase-IV.
(PDF)

Figure S7 **Exponential fits to colony-area growth from the microscopy assay.** Examples of typical microcolony-area data extracted from microscopy images (blue points) fitted with a two-parameter function (blue line) y = A*exp(B*x) to estimate the instantaneous growth rates of single cells.
(PDF)

Figure S8 **Single-cell growth-rate distributions from repeated batch cultures.** (a–d) Repeated experiments show similar dynamics of the distributions of Fig. 3.
(PDF)

Figure S9 **Mean and Standard deviation of growth rates for repeated batch cultures.** (a) Repeated experiments show qualitatively similar fluctuations in the population-average growth-rate as a function of time as in Fig. 4a. The black curve is the data from Fig. 4a. All the measurements exhibit periods of decrease in the mean growth rate on short timescales, suggesting that growth rate is not stably inherited. The green, blue, red and orange traces are the means of the distributions in Figs. S6a-d, respectively. Note that the starting point of each measurement depends on the adaptation dynamics of each batch which is highly variable (see Methods). The population must be growing exponentially first to allow a meaningful measurement of growth rate. (b) Large fluctuations in the standard deviation are seen in all five repeated experiments of (a). The black curve is the data from Fig. 4b. Colors correspond to the same experiments as in (a).
(PDF)

Figure S10 **Fraction of exponentially growing cells in repeated experiments.** Repeated experiments showing a fluctuating fraction of exponentially growing cells in the population similar to Fig. 4c. While the total fraction approaches 1, the rate of convergence is very slow considering the rate of dilution of the batch. The black curve is the same experiment shown in Fig. 4c, and the colors correspond to the same experiments as in Fig. S9.
(PDF)

Figure S11 **Wild-type growth rate distributions.** (a) Control measurements were made on wild-type (YPH499) cells grown in minimal glucose medium with complete amino acids. The time axis indicates hours after switch from galactose to glucose in the batch culture, and was chosen to be in the range of the other batch experiments. (b) The mean growth rate increases by a factor of 1.1 in the course of the measurement, which lasts 50 generations (in comparison with the data from figure 4a, which fluctuate by a factor of 2.4).
(PDF)

Figure S12 **Analysis of microscopy images.** (a) a typical image with the black line surrounding each colony marking the edge used to determine the colony area. Scale bar-50 microns (75 pixels). (b) The line shows the best fit (y = 1.59 exp(0.216*t) to manual cell count (blue circles) and colony area from the automated image analysis (red x). Both cell count and colony area lead to similar estimate of the exponential growth. Colony area is scaled by a single cell area of 45 pixels.
(PDF)

Figure S13 **Error bars of growth-rate distributions.** Representative histograms from the same experiment as in Fig. 3 showing the error-bars on each bin and confirming the significance of the distributions. These distributions contain the smallest number of data points, and therefore show the largest error of all the data sets.
(PDF)
