# Supplementary figures and images for: Population Dynamics of Metastable Growth-Rate Phenotypes
Source: PLoS One. 2013 Dec 2;8(12):e81671. doi: 10.1371/journal.pone.0081671 (PMC3847162; doi:10.1371/journal.pone.0081671)

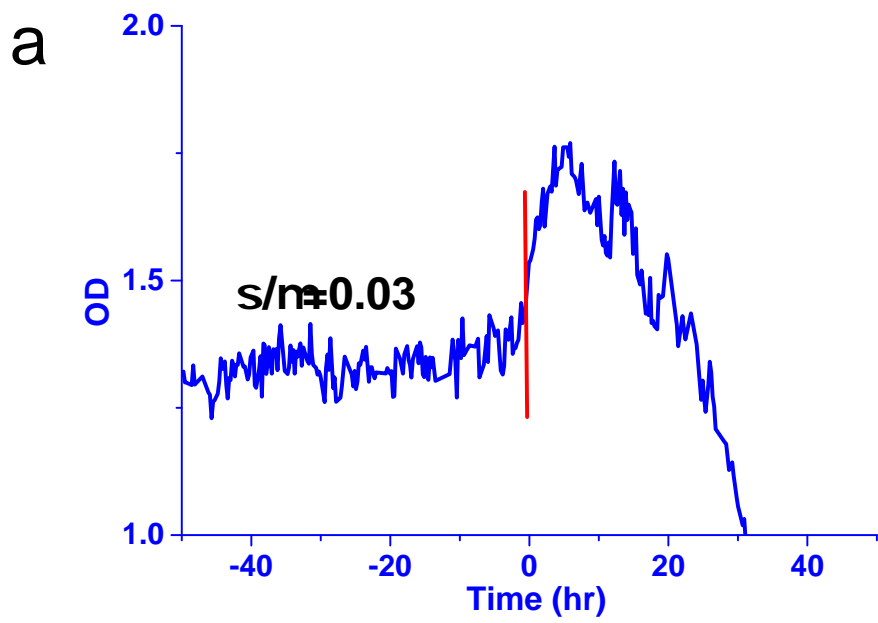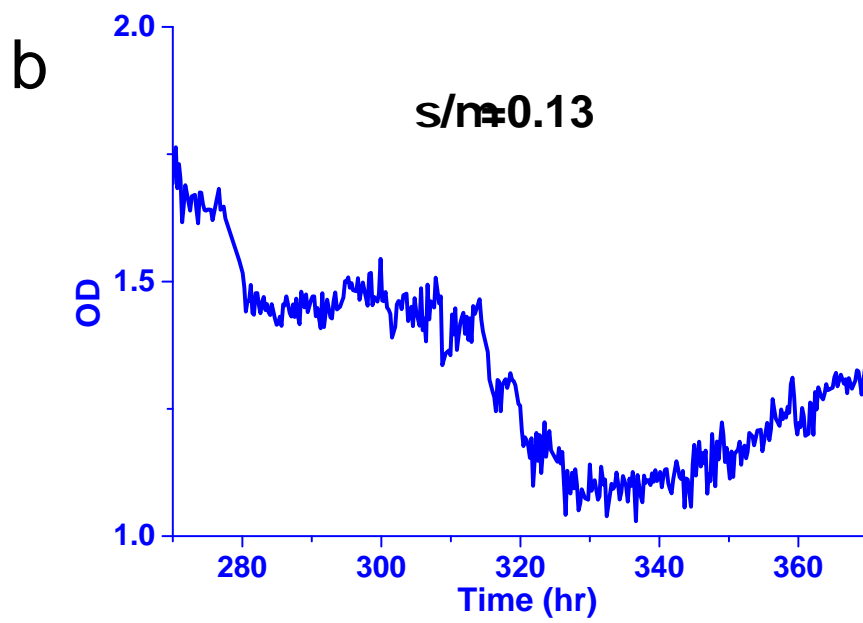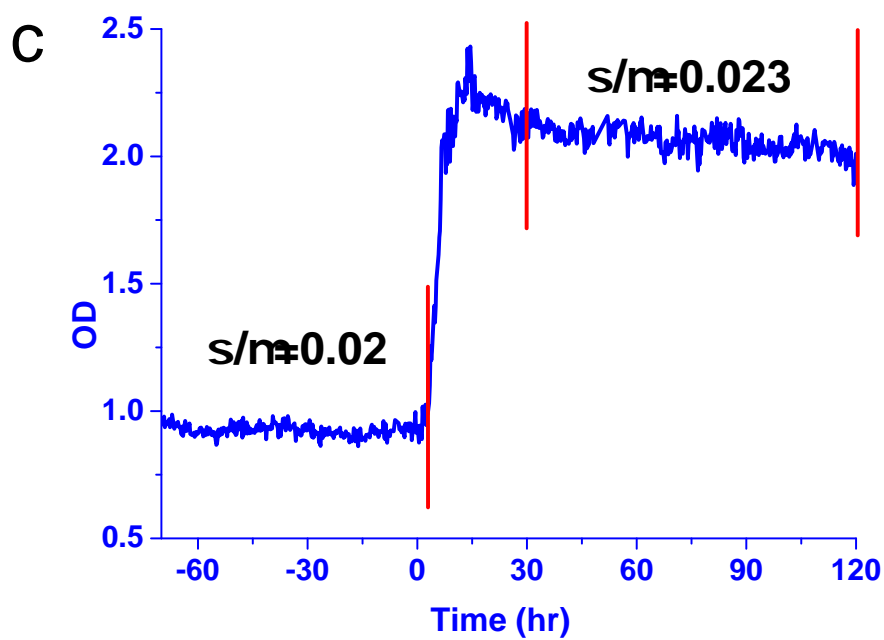

Fig. S1

Supplement: Figure S1 — Chemostat population density fluctuations. (a) The OD of the same chemostat population as in Fig. 1 of the main text, in galactose before switching to glucose at t = 0. (b) Part of phase IV from the same experiment for comparison, showing the significance of the density fluctuations. (c) A population of wild type cells switched from galactose to glucose in the same chemostat apparatus. The σ/μ marked for each region separately, are of the same order as in (a). It shows that the background fluctuations in the chemostat are significantly smaller than the ones observed in phase IV in (b). (PDF) [file pone.0081671.s001.pdf]

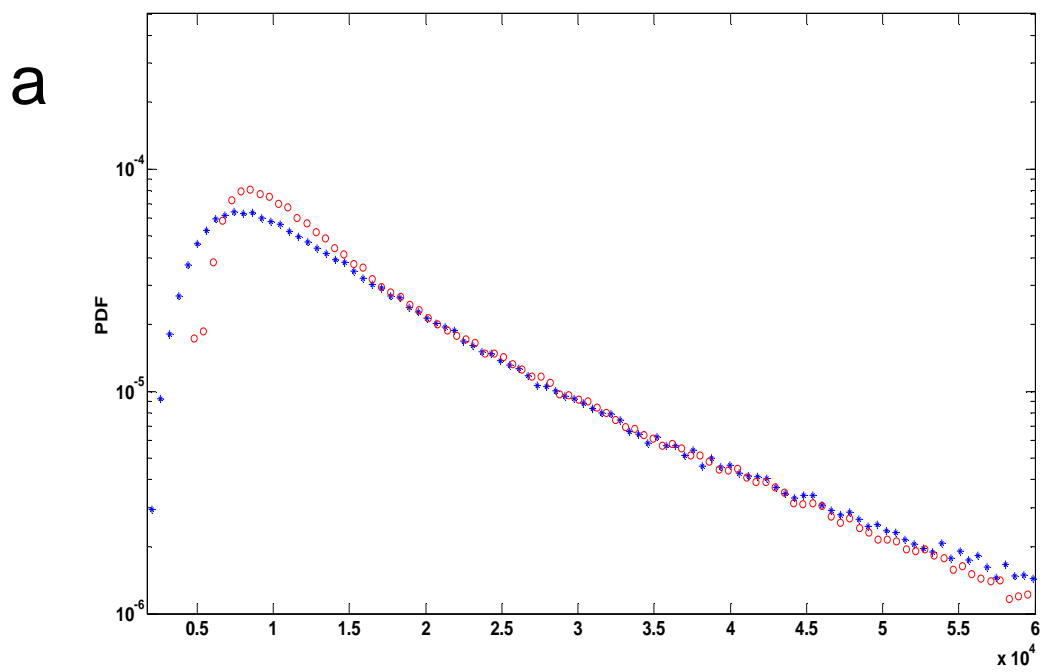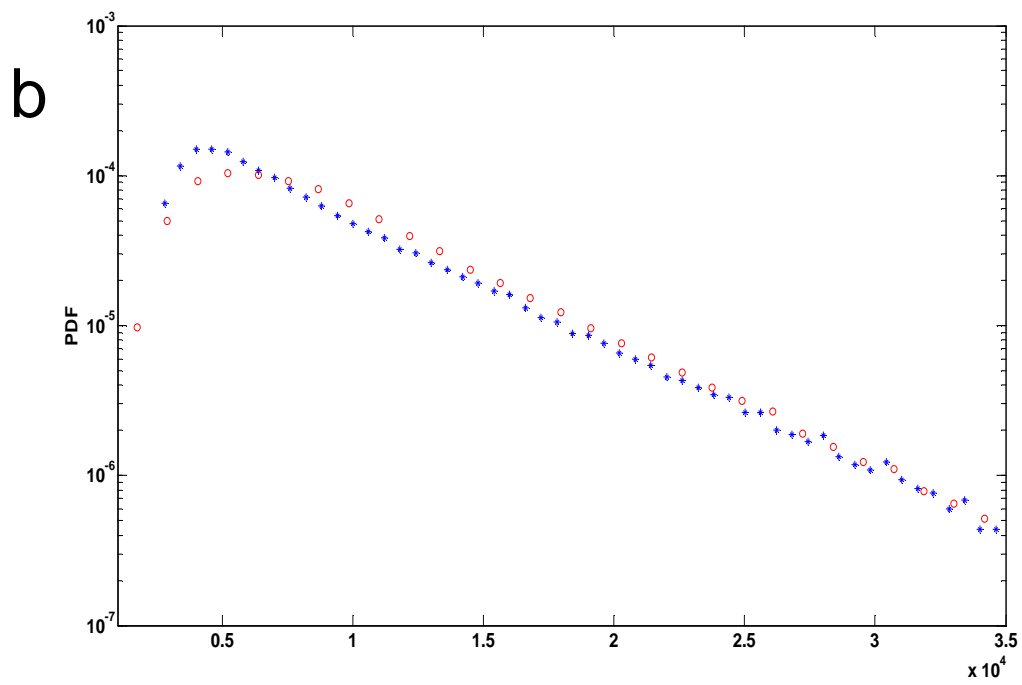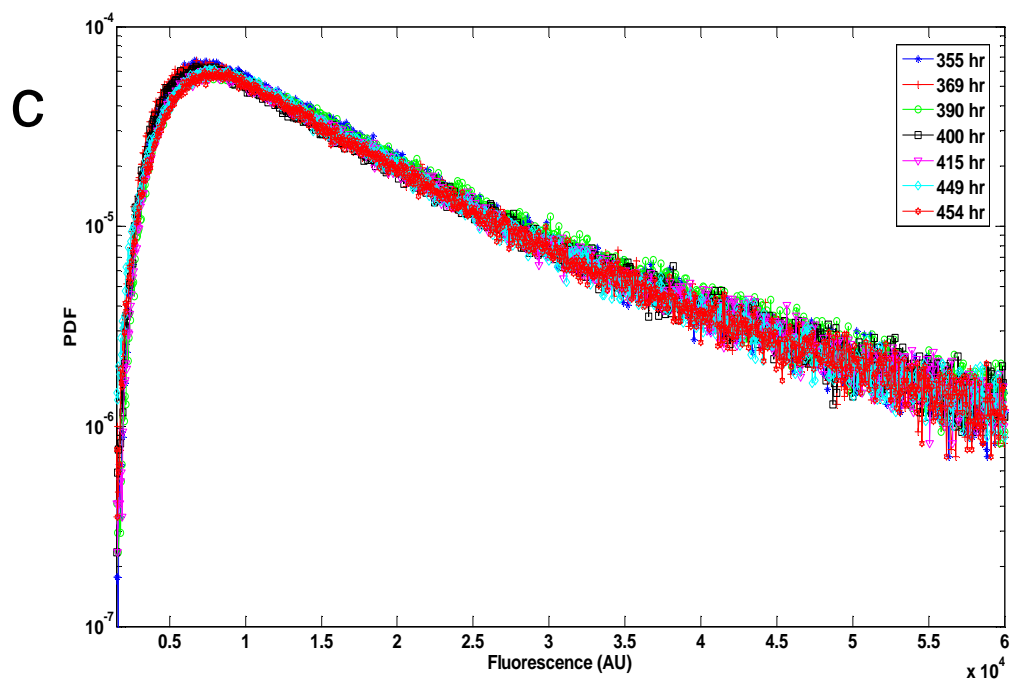

Fig. S2

Supplement: Figure S2 — Stability and accuracy of homemade cell cytometer. (a) and (b) show the comparison of our home-made cytometer data with a commercial flow cytometer (BD LSR-II Analyzer), with agreement between the data sets of >95%. (c) The stability of the home-made cytometer measurements is shown by comparing the HIS3p-GFP distributions of a chemostat population in galactose medium over a period of 100 hours (∼20 generations). (PDF) [file pone.0081671.s002.pdf]

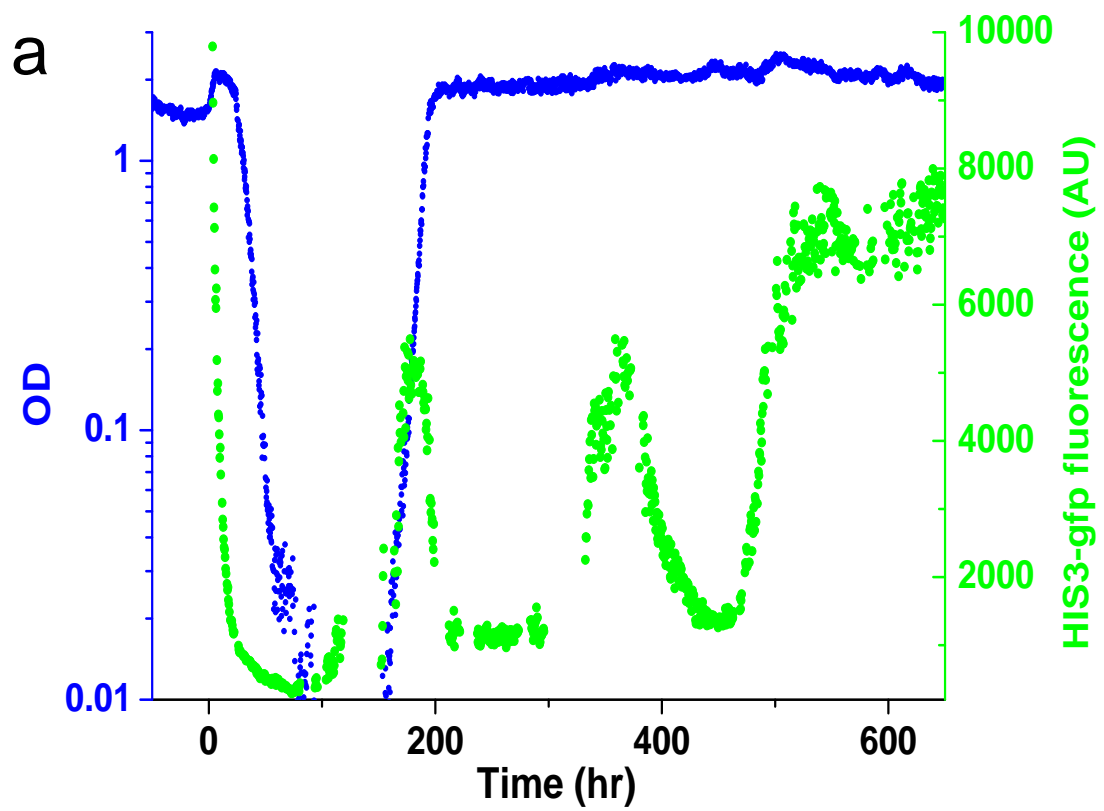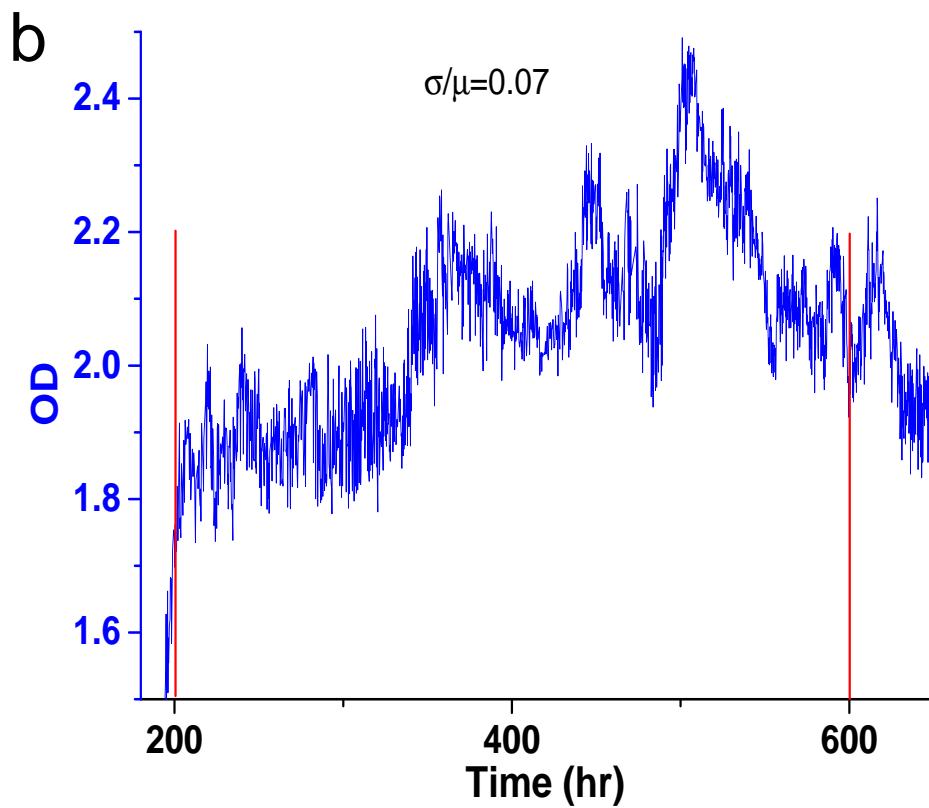

Fig. S3

Supplement: Figure S3 — Mean HIS3-GFP dynamics in a chemostat population. (a) A repeated experiment to the one shown in Fig. 2a. The blue trace is the chemostat optical density as a function of time after switch from galactose medium to glucose medium lacking histidine at t = 0. Note the logarithmic scale. The green trace is the mean fluorescence measurement of HIS3-GFP. The population-average fluorescence was extracted from statistics over single-cell measurements utilizing our home-made cytometer online with the chemostat. (b) The OD in phase IV of the chemostat in (a) on a linear scale between 200 and 600 hours showing significant fluctuations similar to the ones observed in Fig. 1 of the main text. (PDF) [file pone.0081671.s003.pdf]

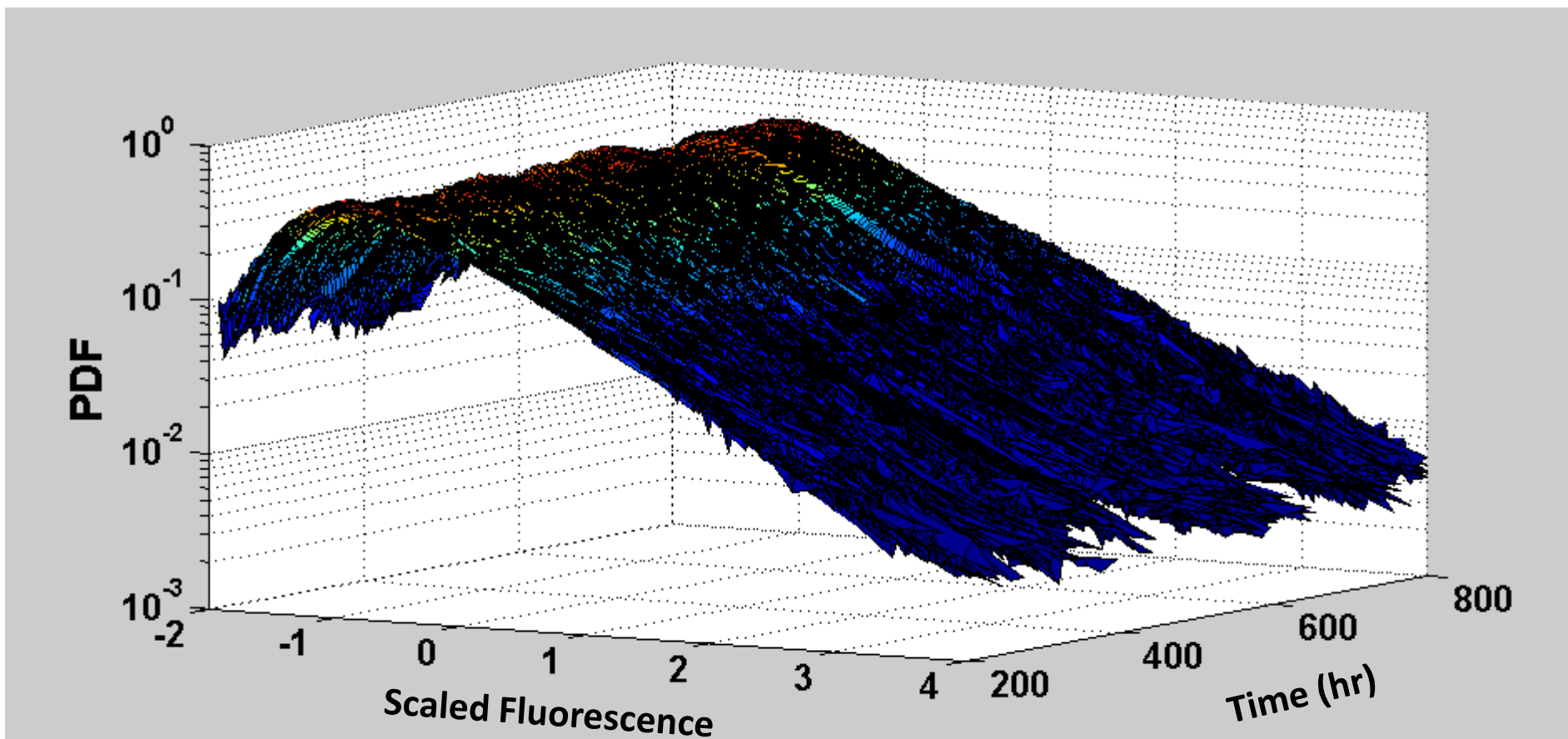

Fig. S4

Supplement: Figure S4 — Scaled HIS3-GFP distributions. Single-cell fluorescence distributions measured from the same chemostat population as in Fig. 1 during phase-IV. All distributions have been scaled by subtracting the mean and dividing by the standard deviation, causing them to collapse onto a similar shape. Note that the mean fluorescence value from this same time period (Fig. 2a) has dynamic fluctuations by more than a factor of three. (PDF) [file pone.0081671.s004.pdf]

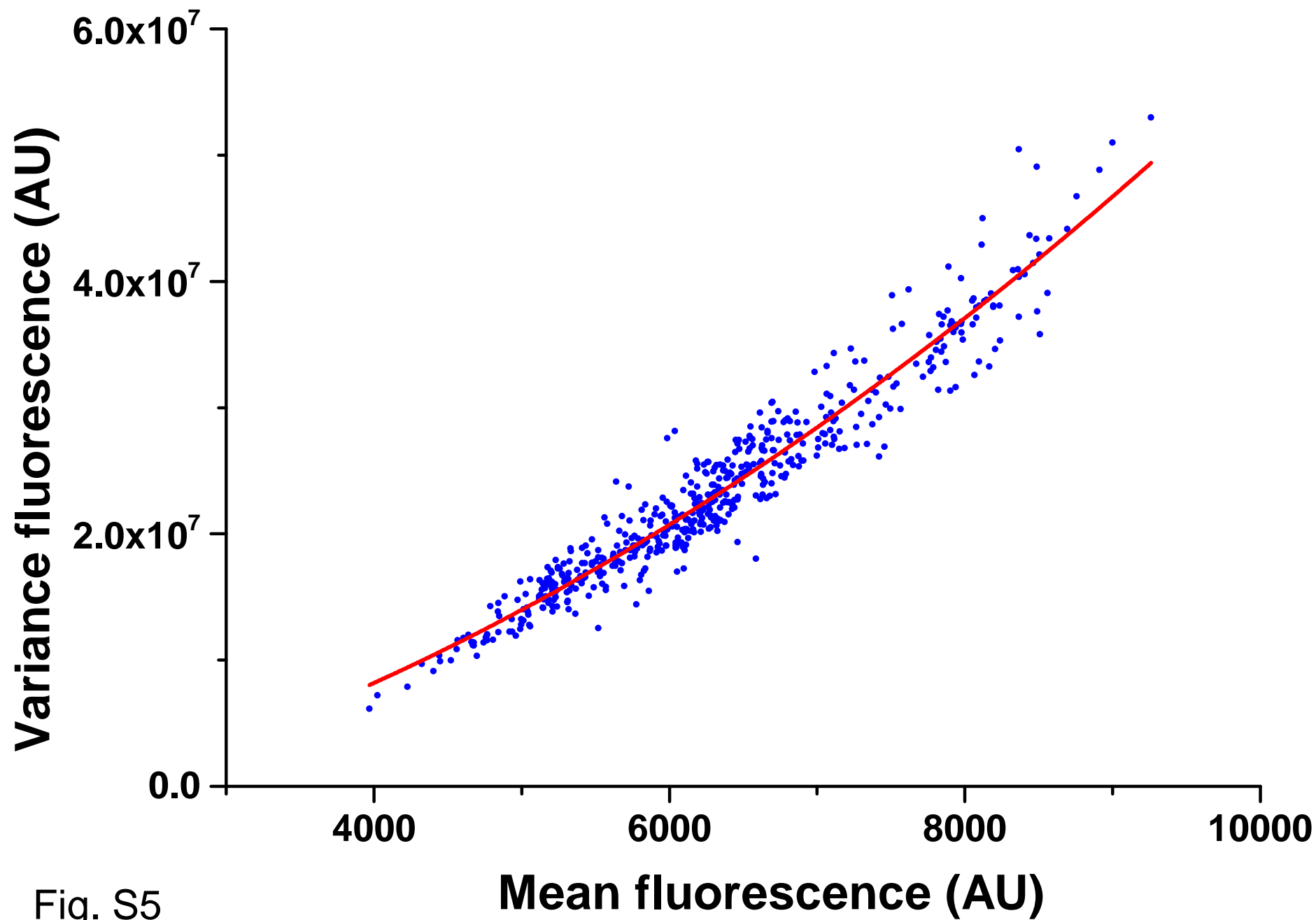

Fig. S5

Supplement: Figure S5 — Variance vs. Mean from HIS3-GFP distributions. Scatter plot of the variance fluorescence versus the mean extracted from single-cell fluorescence distributions for the same populations of cells as in Fig. 2, for the entire range in phase IV as in Fig. S4. The variance is a quadratic function of the mean: red curve, best fit y = −5.39+1478 x+0.48 x2. (PDF) [file pone.0081671.s005.pdf]

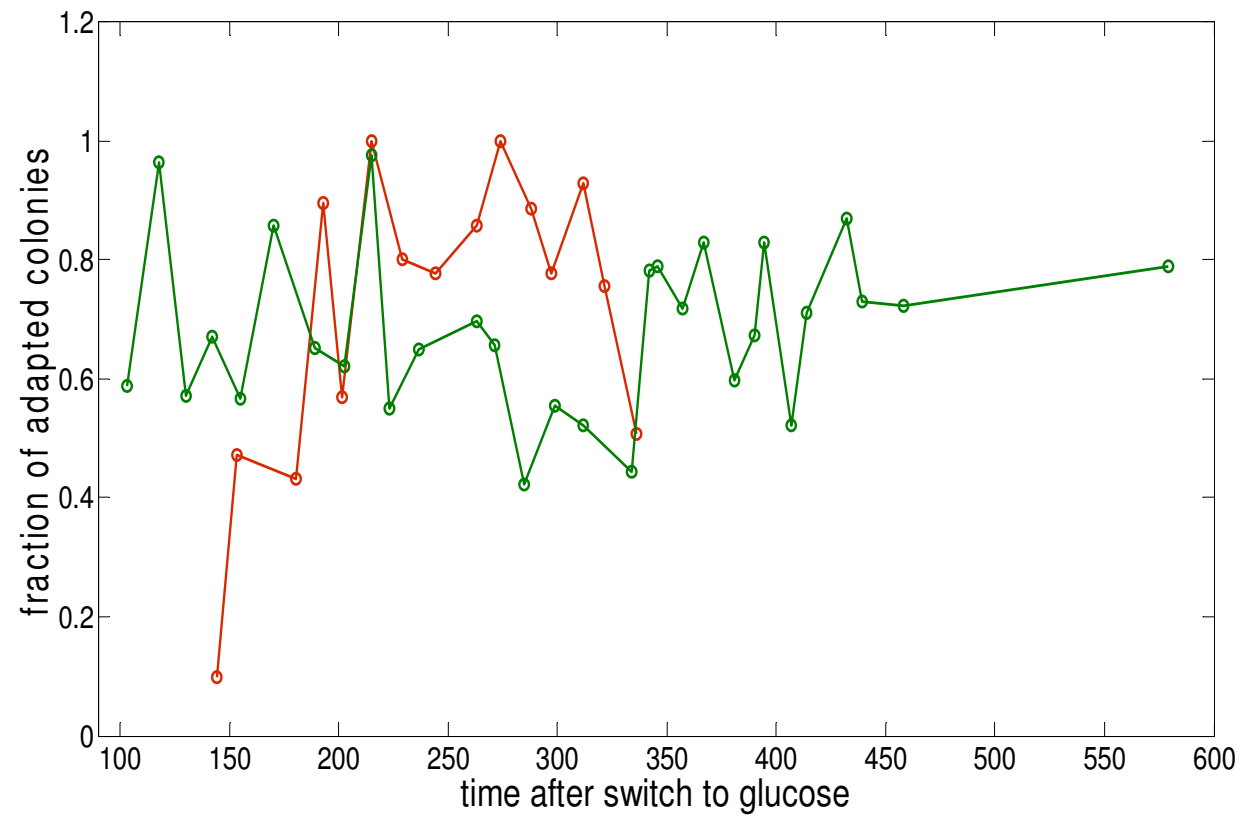

Fig. S6

Supplement: Figure S6 — Fraction of adapted cells in a batch culture. The number of cells that are able to grow a visible colony within 3 days after plating on glu-his agar plates (“fraction adapted”) relative to the number of colonies grown on rich medium plates from serially-diluted batch culture in phase IV, after the switch from galactose to glucose. Comparison with the red curve in Fig. 1 shows qualitatively similar fluctuations in the fraction of adapted cells in a chemostat culture and in batch cultures throughout phase-IV. (PDF) [file pone.0081671.s006.pdf]

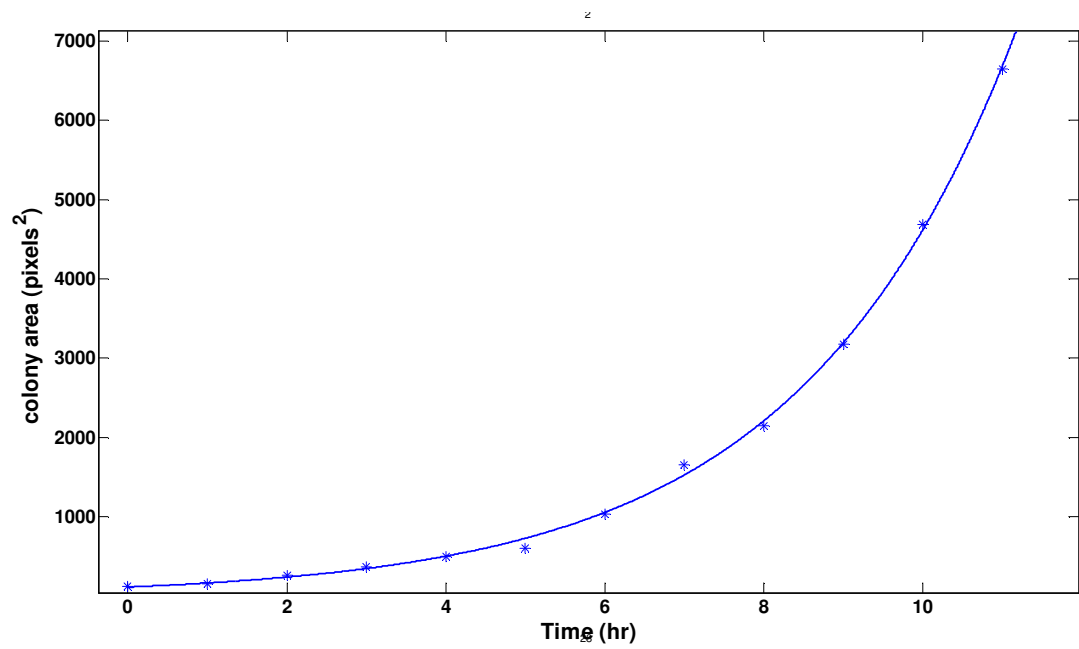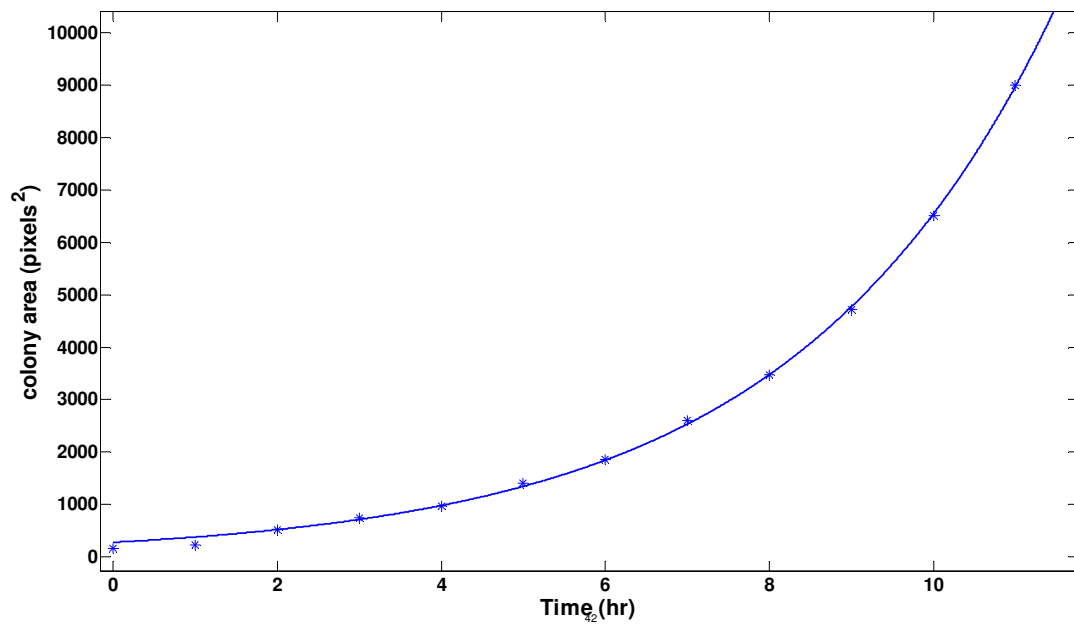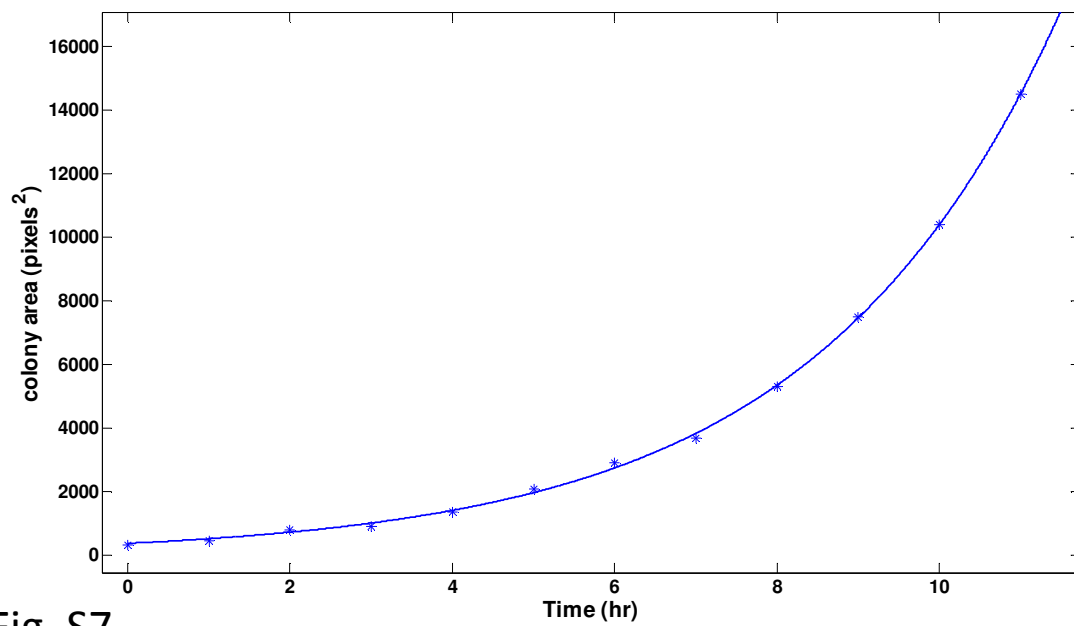

Fig. S7

Supplement: Figure S7 — Exponential fits to colony-area growth from the microscopy assay. Examples of typical microcolony-area data extracted from microscopy images (blue points) fitted with a two-parameter function (blue line) y = A*exp(B*x) to estimate the instantaneous growth rates of single cells. (PDF) [file pone.0081671.s007.pdf]

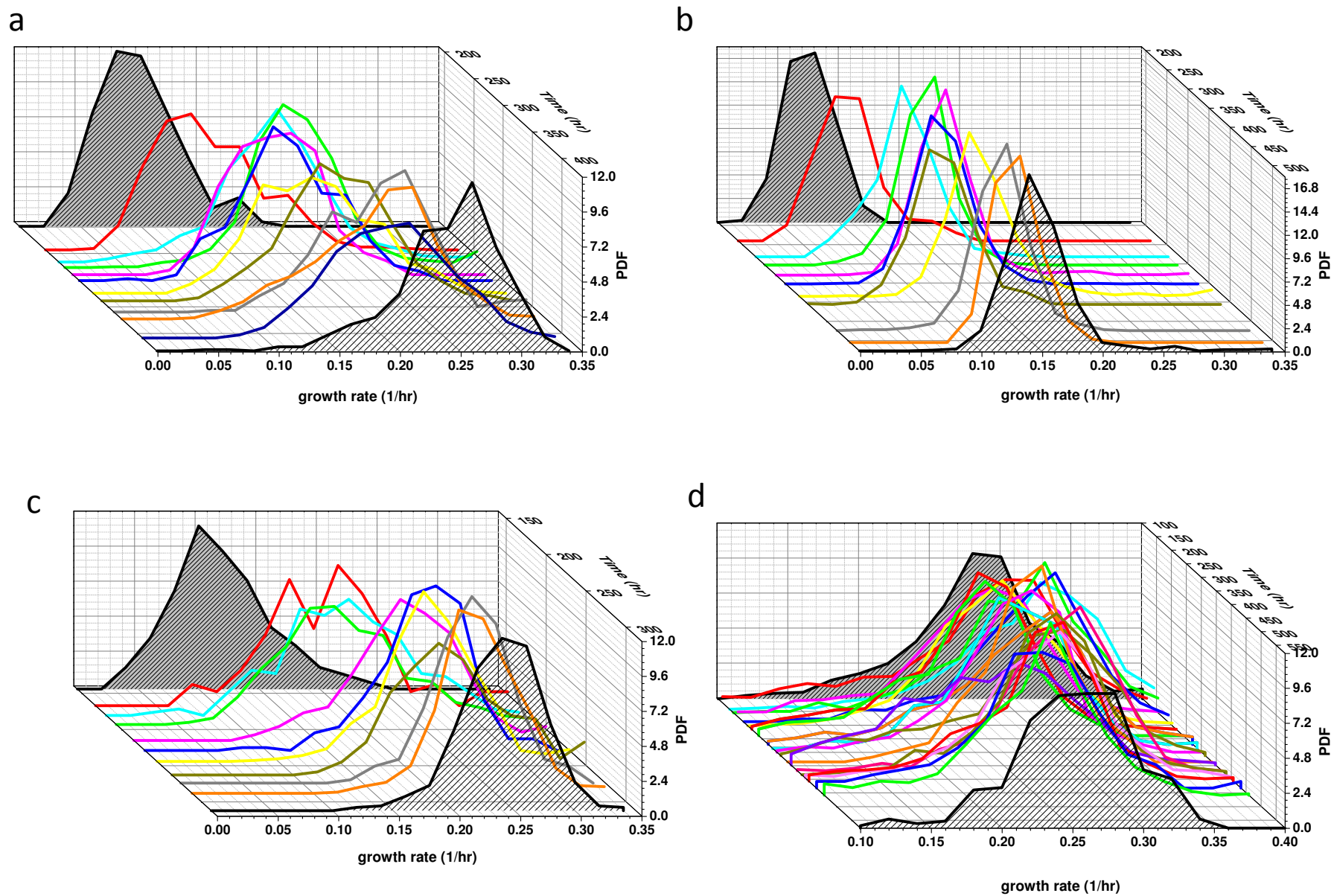

Fig. S8

Supplement: Figure S8 — Single-cell growth-rate distributions from repeated batch cultures. (a–d) Repeated experiments show similar dynamics of the distributions of Fig. 3. (PDF) [file pone.0081671.s008.pdf]

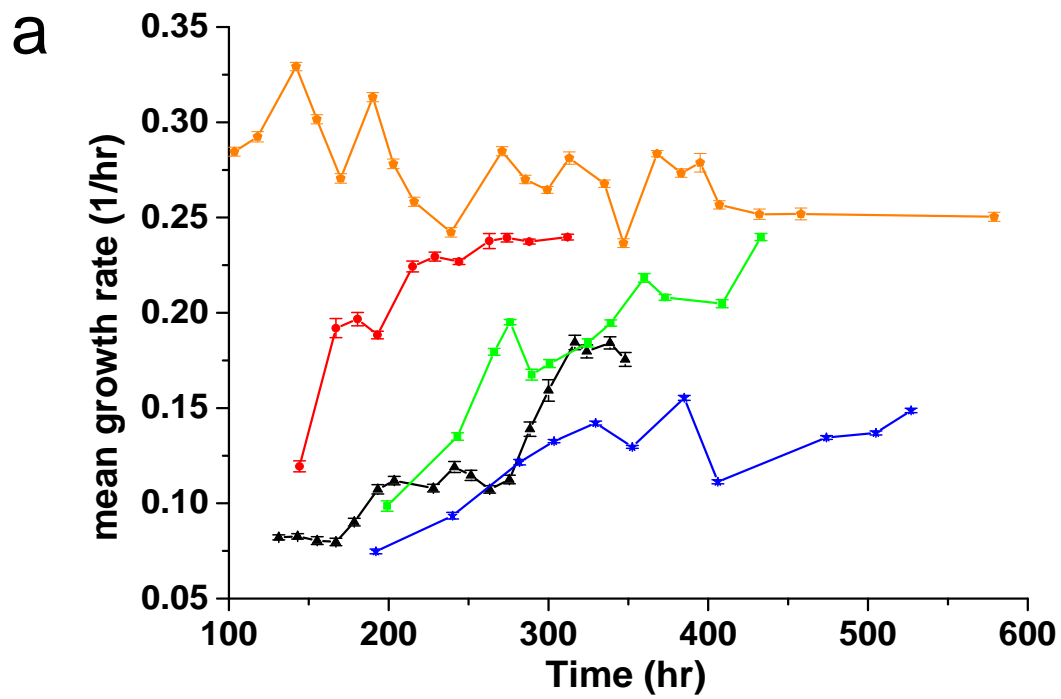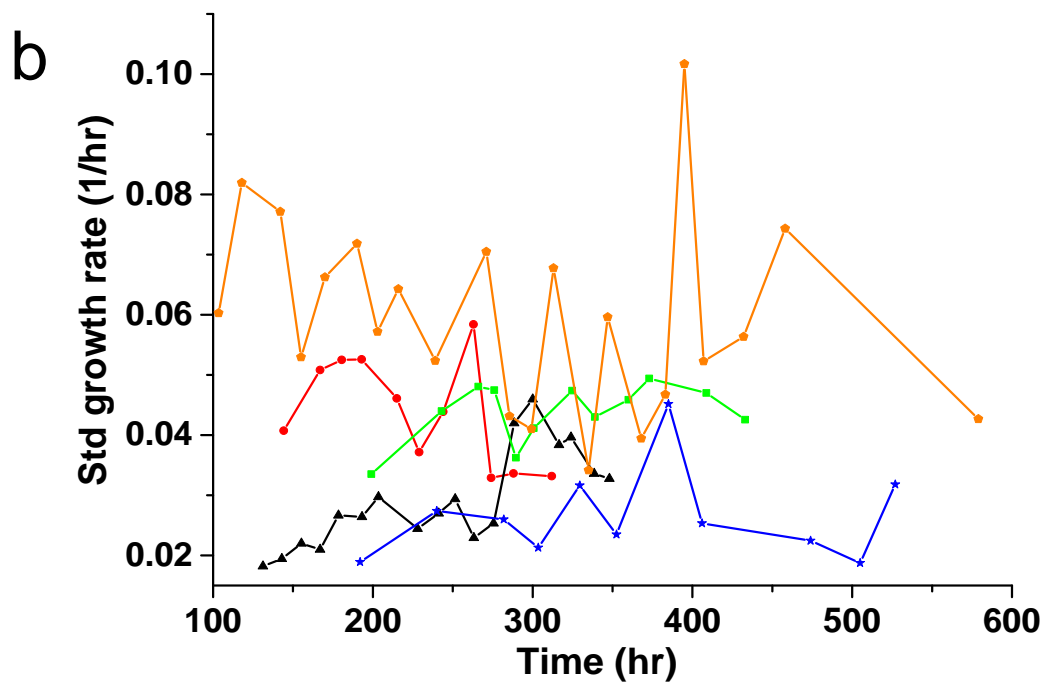

Fig. S9

Supplement: Figure S9 — Mean and Standard deviation of growth rates for repeated batch cultures. (a) Repeated experiments show qualitatively similar fluctuations in the population-average growth-rate as a function of time as in Fig. 4a. The black curve is the data from Fig. 4a. All the measurements exhibit periods of decrease in the mean growth rate on short timescales, suggesting that growth rate is not stably inherited. The green, blue, red and orange traces are the means of the distributions in Figs. S6a-d, respectively. Note that the starting point of each measurement depends on the adaptation dynamics of each batch which is highly variable (see Methods). The population must be growing exponentially first to allow a meaningful measurement of growth rate. (b) Large fluctuations in the standard deviation are seen in all five repeated experiments of (a). The black curve is the data from Fig. 4b. Colors correspond to the same experiments as in (a). (PDF) [file pone.0081671.s009.pdf]

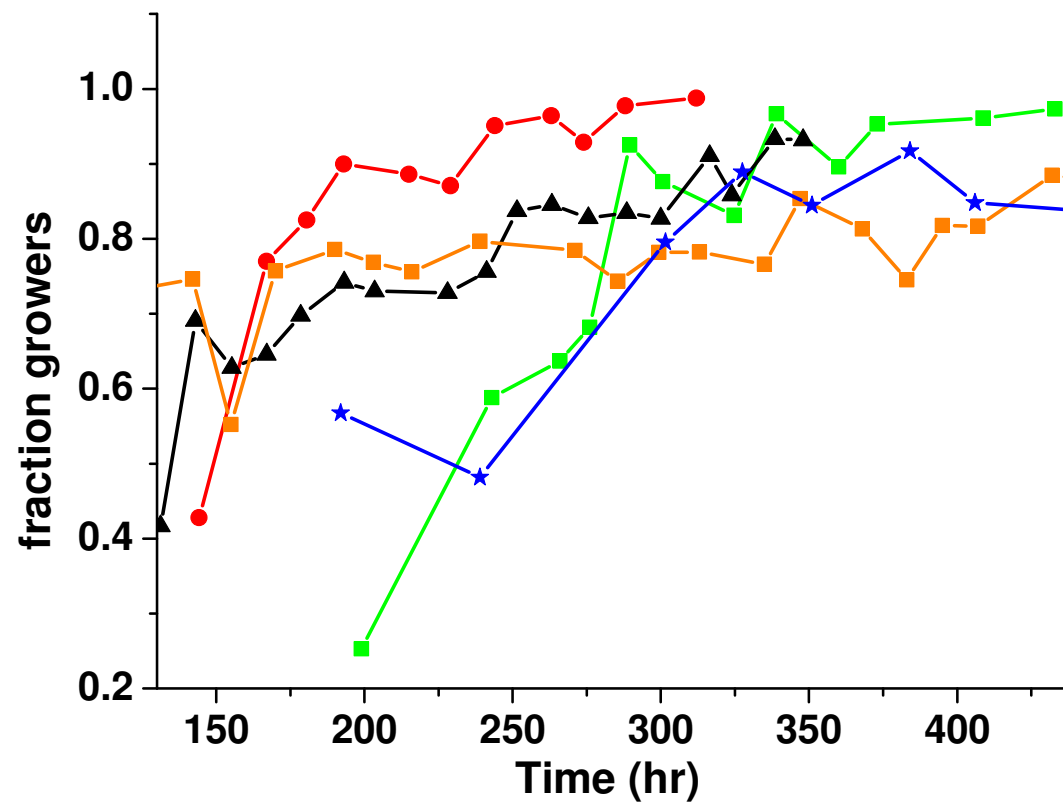

Fig. S10

Supplement: Figure S10 — Fraction of exponentially growing cells in repeated experiments. Repeated experiments showing a fluctuating fraction of exponentially growing cells in the population similar to Fig. 4c. While the total fraction approaches 1, the rate of convergence is very slow considering the rate of dilution of the batch. The black curve is the same experiment shown in Fig. 4c, and the colors correspond to the same experiments as in Fig. S9. (PDF) [file pone.0081671.s010.pdf]

a

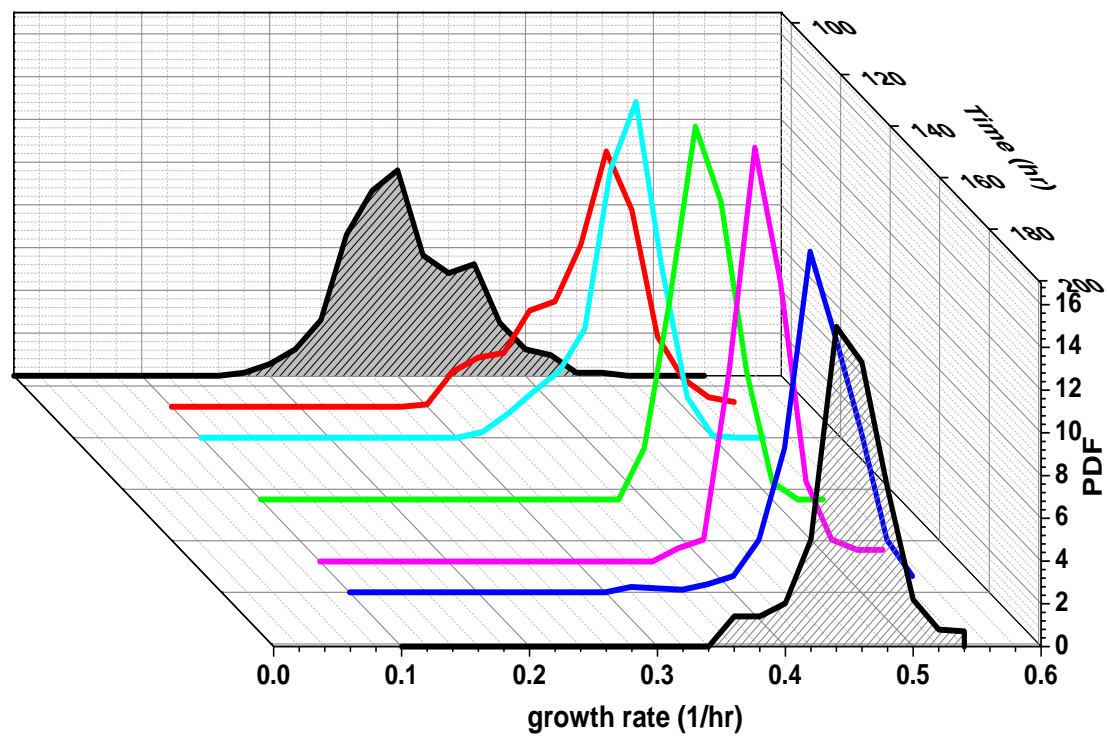

b

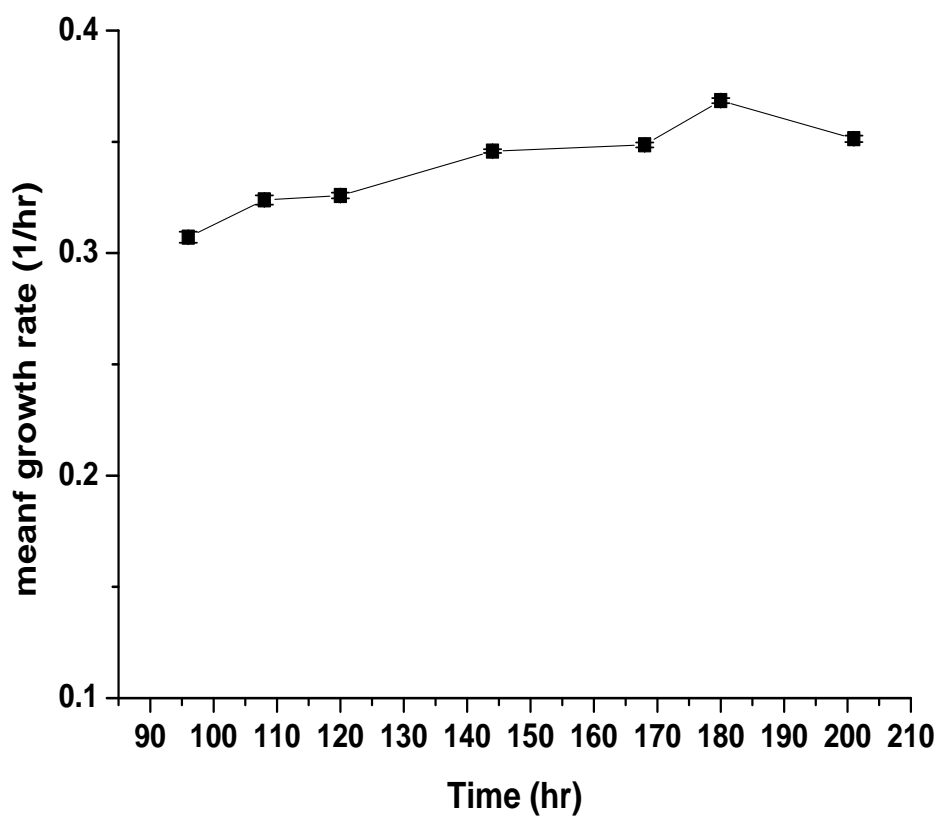

Fig. S11

Supplement: Figure S11 — Wild-type growth rate distributions. (a) Control measurements were made on wild-type (YPH499) cells grown in minimal glucose medium with complete amino acids. The time axis indicates hours after switch from galactose to glucose in the batch culture, and was chosen to be in the range of the other batch experiments. (b) The mean growth rate increases by a factor of 1.1 in the course of the measurement, which lasts 50 generations (in comparison with the data from figure 4a, which fluctuate by a factor of 2.4). (PDF) [file pone.0081671.s011.pdf]

a

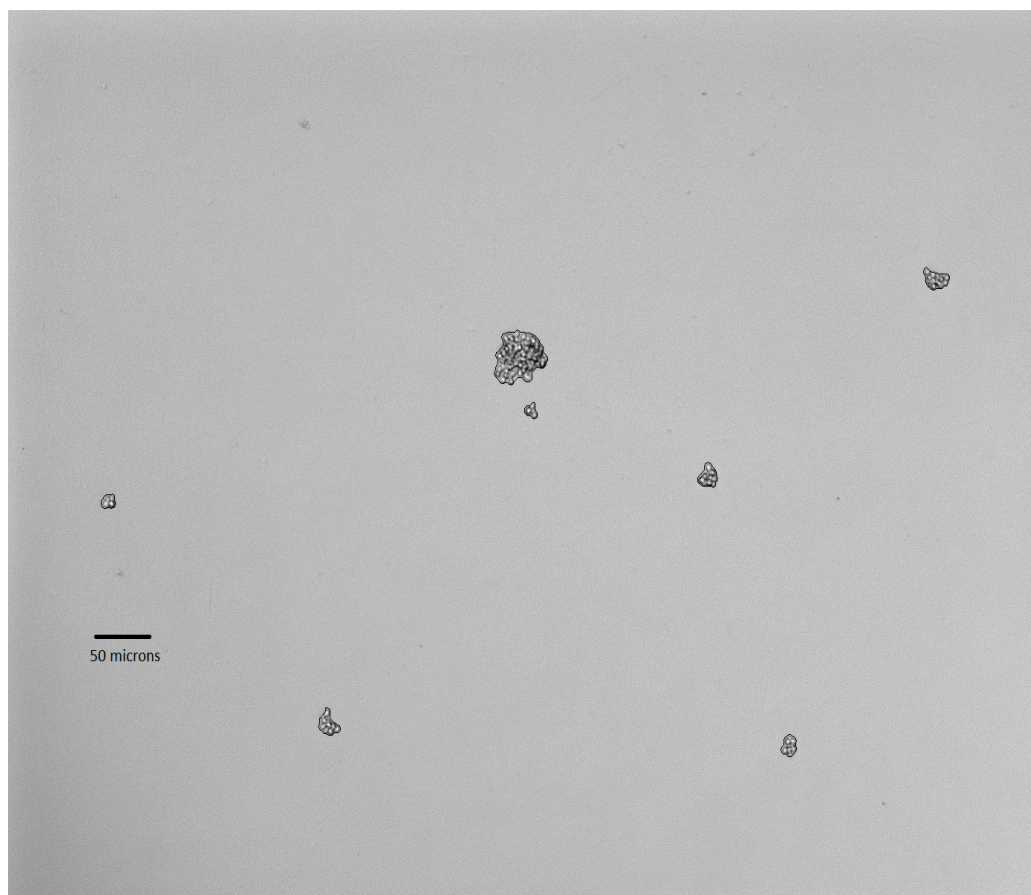

b

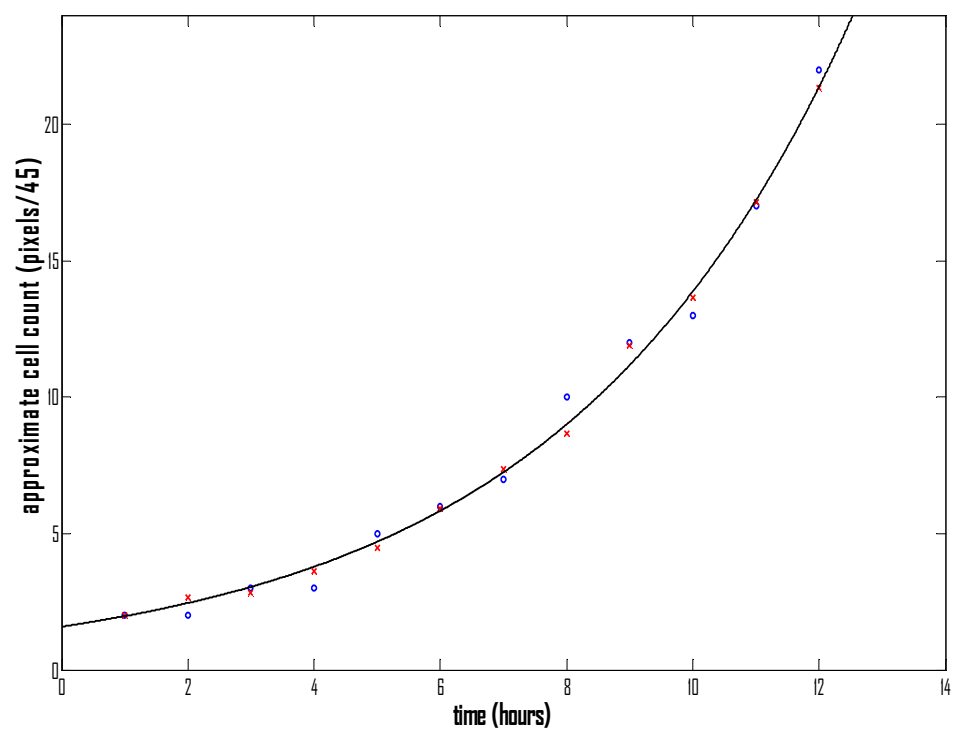

Fig. S12

Supplement: Figure S12 — Analysis of microscopy images. (a) a typical image with the black line surrounding each colony marking the edge used to determine the colony area. Scale bar-50 microns (75 pixels). (b) The line shows the best fit (y = 1.59 exp(0.216*t) to manual cell count (blue circles) and colony area from the automated image analysis (red x). Both cell count and colony area lead to similar estimate of the exponential growth. Colony area is scaled by a single cell area of 45 pixels. (PDF) [file pone.0081671.s012.pdf]

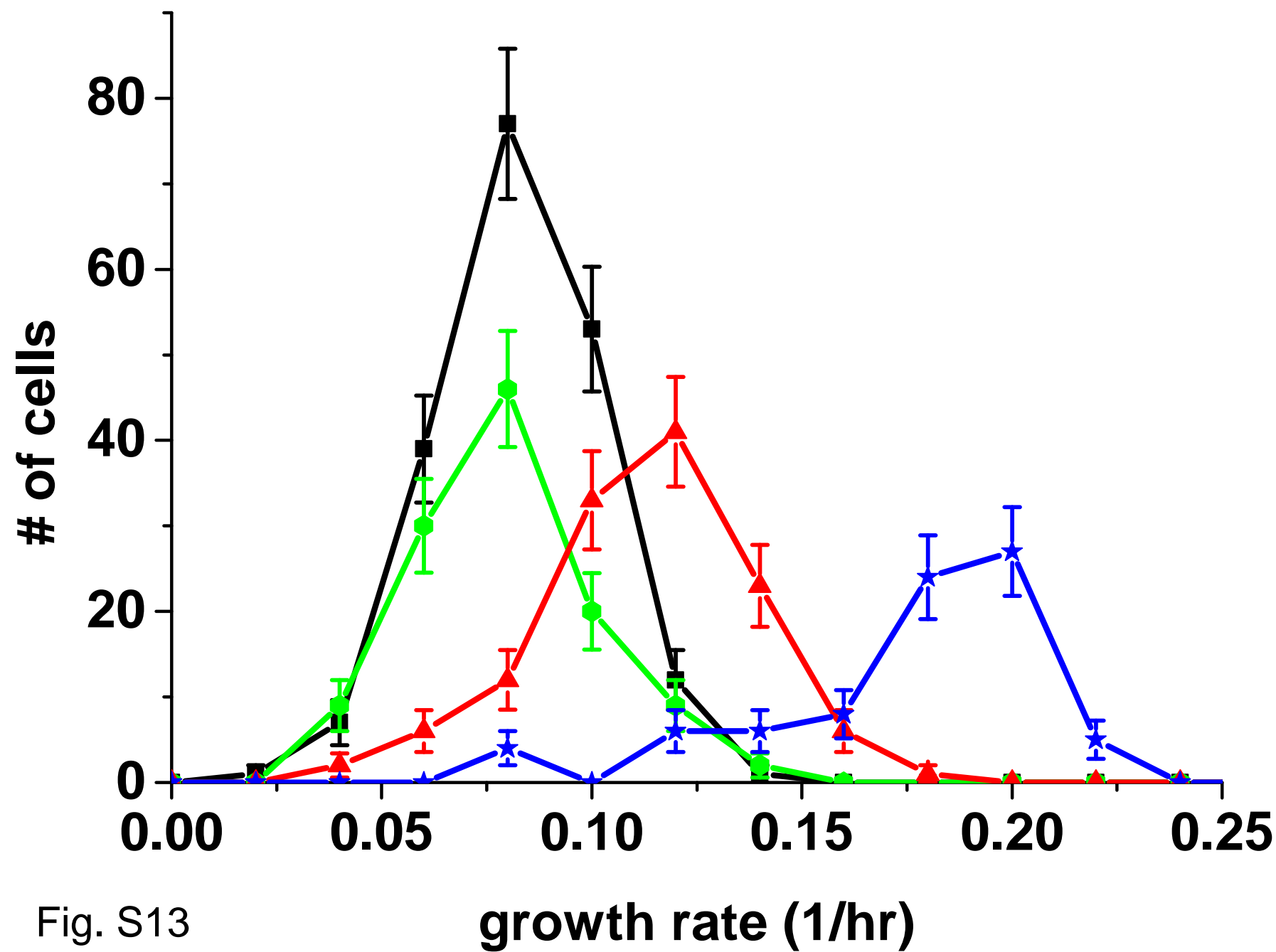

Fig. S13

Supplement: Figure S13 — Error bars of growth-rate distributions. Representative histograms from the same experiment as in Fig. 3 showing the error-bars on each bin and confirming the significance of the distributions. These distributions contain the smallest number of data points, and therefore show the largest error of all the data sets. (PDF) [file pone.0081671.s013.pdf]
